# Study of the Bcl-2 Interactome by BiFC Reveals Differences in the Activation Mechanism of Bax and Bak

**DOI:** 10.3390/cells12050800

**Published:** 2023-03-03

**Authors:** Óscar Gonzalo, Andrea Benedi, Laura Vela, Alberto Anel, Javier Naval, Isabel Marzo

**Affiliations:** Department Biochemistry, Molecular and Cell Biology, Faculty of Science, University of Zaragoza, 50009 Zaragoza, Spain

**Keywords:** Bcl-2 family, protein–protein interactions, apoptosis, BH3-mimetics, Bimolecular Fluorescence Complementation

## Abstract

Evasion of apoptosis is one of the hallmarks of cancer cells. Proteins of the Bcl-2 family are key regulators of the intrinsic pathway of apoptosis, and alterations in some of these proteins are frequently found in cancer cells. Permeabilization of the outer mitochondrial membrane, regulated by pro- and antiapoptotic members of the Bcl-2 family of proteins, is essential for the release of apoptogenic factors leading to caspase activation, cell dismantlement, and death. Mitochondrial permeabilization depends on the formation of oligomers of the effector proteins Bax and Bak after an activation event mediated by BH3-only proteins and regulated by antiapoptotic members of the Bcl-2 family. In the present work, we have studied interactions between different members of the Bcl-2 family in living cells via the BiFC technique. Despite the limitations of this technique, present data suggest that native proteins of the Bcl-2 family acting inside living cells establish a complex network of interactions, which would fit nicely into “mixed” models recently proposed by others. Furthermore, our results point to differences in the regulation of Bax and Bak activation by proteins of the antiapoptotic and BH3-only subfamilies. We have also applied the BiFC technique to explore the different molecular models proposed for Bax and Bak oligomerization. Bax and Bak’s mutants lacking the BH3 domain were still able to associate and give BiFC signals, suggesting the existence of alternative surfaces of interaction between two Bax or Bak molecules. These results agree with the widely accepted symmetric model for the dimerization of these proteins and also suggest that other regions, different from the α6 helix, could be involved in the oligomerization of BH3-in groove dimers.

## 1. Introduction

The key event in the intrinsic pathway of apoptosis is mitochondrial outer membrane permeabilization and the release of apoptogenic proteins. The effectors of this critical process are Bax and Bak, two proapoptotic proteins belonging to the Bcl-2 family. Thus, the presence of at least one of these two proteins is required for cell death through the intrinsic pathway initiated by many apoptotic stimuli [1]. The Bcl-2 family also comprises a group of antiapoptotic proteins that keep proapoptotic members in check to avoid accidental activation of Bax and Bak. Finally, the family includes a subset of proapoptotic proteins, containing only the BH3 homology domain (the BH3-only group), which act as cell damage sensors and trigger Bax and Bak activation. The way BH3-only proteins activate multidomain proapoptotic proteins has been a matter of controversy in the field. The recent approval by the FDA of Venetoclax, a BH3-mimetic Bcl-2 inhibitor, for the treatment of Chronic Lymphocytic Leukemia [2] has fueled interest in understanding the mechanisms that regulate the interactions between these proteins. Initially, two opposed models were proposed, the “displacement” (or “indirect”) and the “direct” model [3]. One of the discrepancies between both models was how BH3-only proteins triggered Bax and Bak oligomerization. The “displacement or indirect” model postulated that active Bax and Bak are blocked by antiapoptotic members of the Bcl-2 family until they are bound and neutralized by BH3-only proteins in response to proapoptotic signals [4]. By contrast, in the “direct model”, BH3-only proteins were proposed to bind and activate Bax and Bak. Initially, only tBid and Bim [5] were included in this group of “activators”, but later, also Puma [6] and Noxa [7] were also suggested to belong to this category. In this model, antiapoptotic proteins inhibit mitochondrial permeabilization by blocking activator BH3-only proteins. A second group of BH3-only proteins, the “sensitizers”, free the activators by releasing them from the restraint of antiapoptotic proteins. Free BH3-only activators can then bind to and activate Bax and Bak through a conformational change that leads to oligomerization and pore formation. Some genetic studies in mice and immunoprecipitation data seemed to exclude a direct interaction between BH3-only and multidomain proapoptotic proteins [4], while other reports suggested that these interactions could be necessary for the proapoptotic function of some BH3-only proteins [8]. The controversy was also fed by the fact that direct activation of Bax and Bak by BH3-only proteins remained difficult to detect in living cells. Immunoprecipitation analysis of the interactions between Bcl-2 family members has habitually failed to detect an association of BH3-only proteins with Bax or Bak. By contrast, in vitro studies with purified proteins and peptides have provided evidence that some BH3-only proteins can bind to Bax [9,10] and Bak [6,7]. It is important to note that experimental conditions in some of these studies may not truly reproduce the interactions that could occur between full-length proteins in a cellular context. Recently, we were able to visualize the interactions of Bim, Puma, or Noxa with Bax and Bak in living cells via the BiFC (Bimolecular Fluorescence Complementation) technique [11]. Additionally, the results of this previous work outlined the complexity of the Bcl-2 family interactome, supporting more recent models, such as the “embedded together” and the “unified” models, that include features of both the direct and indirect models [12].

An important, unsolved question concerning BH3-only activators is how these proteins would bind to Bax and Bak. Transient binding of activator BH3-only proteins to the hydrophobic groove of Bax and Bak has been proposed to provoke the activation of Bax and Bak [7,13] by causing the exposure of their BH3 domain followed by dimerization. Alternatively, other authors have reported that activator BH3-only proteins interact with Bax in a “rear pocket” formed by α1 and α6 helices triggering the release of the C-terminal helix [14,15,16]. These apparent discrepancies could reflect variations in the mechanism of activation of Bak and Bax. Although both proteins function as effectors of mitochondrial outer membrane permeabilization, differences in structure and localization could indicate that they are not completely equivalent. In healthy cells, Bax is usually a cytosolic protein that translocates to mitochondria in the early steps of apoptosis. In contrast, Bak is constitutively located at mitochondria, where its α9 helix acts as an anchor to the outer membrane. This helix could be occluded in a hydrophobic pocket in an inactive cytosolic Bax [17,18].

Many models for mitochondrial permeabilization postulate that activated Bax and Bak oligomerize to form pores in the outer mitochondrial membrane. The disposition of monomers in these pores is also a matter of controversy [19]. According to the asymmetric model, the BH3 domain of an activated molecule inserts itself into the rear pocket of another one, triggering exposure of the BH3 domain of the second monomer and expansion of the oligomer. Alternatively, a symmetric model proposes that dimers are formed by the insertion of the BH3 domain of one molecule into the hydrophobic pocket, “BH3-in-groove”, of other molecules and dimers associate in high-order oligomers by interacting through the “rear” surface. Identification of this second interface, involved in the oligomerization of BH3-in-groove dimers, remains an open question in the field, and different regions of Bax and Bak have been proposed to participate in this process. Contact between the α6 helices of two dimers has been demonstrated by the use of cysteine mutants and a disulfide bridging [20,21]. However, the α3/α5 surface [22] and the α9 helix [23,24] have been proposed to mediate the association of symmetric dimers in high-order oligomers. More recently, a new model of disordered dimer clusters has been described to explain Bak oligomerization [25].

Despite some limitations, Bimolecular Fluorescence Complementation (BiFC) is a useful tool for the detection of protein interactions in a cellular context, especially for transient and weak interactions, which are difficult to detect via other methods [26]. Using this technique, we have been able to detect interactions between some members of the Bcl-2 family, including interactions of BH3-only proteins with Bax and Bak [11]. We have now extended our studies to interactions with Bcl-2 antiapoptotic proteins and to the study of Bax and Bak dimerization. Our present results seem to confirm that the BH3-only proteins, Bim, Puma, and Noxa, can bind to Bax and Bak, although with different affinities. According to our present results, Bim is a better activator of Bax than Puma or Noxa. However, Noxa displayed the strongest association with Bak. Furthermore, our results indicate that both the BH3-only and multidomain proapoptotic subset can be blocked by antiapoptotic proteins, pointing to a mixed or “unified” model for the activation of Bax and Bak, proposed by some authors. Finally, using different mutant forms of Bax and Bak, we have explored the roles of BH3, H6α, and H1α regions in the interactions with other proteins of the family and in the dimerization processes. Our results would suggest that several interfaces are involved in the oligomerization of both Bak and Bax proteins.

## 2. Materials and Methods

### 2.1. Construction of pBiFC and pBabe Vectors

The coding sequences for human Bcl-2, Bcl-X_L_, Mcl-1, Bim, Puma, Noxa, Bax, Bak, and TOM20 were subcloned by standard PCR strategies [11] into pBiFC plasmids modified to contain the sequences coding for VN (amino acids 1–173) or VC (amino acids 155–238) fragments of Venus protein and the appropriate linker sequence fused to each protein at the N-terminus. VN fusions contain the sequence of HA tag and VC fusions that of Flag tag (Supplementary Figure S1). For multicolor BiFC experiments, the cDNA of selected proteins of the Bcl-2 family was subcloned into pBiFC vectors containing the amino- or carboxy-fragment of cerulean protein (CN and CC, respectively). Deletions of either the BH3 domain or the α1 helix of Bax and Bak were generated by PCR overlap, as previously described [11]. Standard site-directed mutagenesis was performed to generate BaxK21E/D33A/W139A/D146A/R147A and BakH164A mutants. Enzymes used for mutagenesis included AccuPrime *Pfx* SuperMix (Invitrogen) and *Dpn*I (Fisher BioReagents) digestion. Human Bax and Bak cDNAs (and the corresponding mutated forms) were also subcloned into pBabe plasmid within the appropriated restriction sites (*Eco*RI/*Sal*I for pBabe-Bax and *Eco*RI/*Bam*HI for pBabe-Bak). Sequences of constructs are provided in the Supplementary materials.

### 2.2. Cell Lines

Human HeLa cervix adenocarcinoma and HCT116 Bax^−/−^ colon carcinoma (gently provided by Dr. Julián Pardo, IIS-Aragon) and MiaPaca2 pancreatic carcinoma cells were routinely cultured at 37 °C in DMEM medium supplemented with 10% FBS, L-glutamine, and penicillin/streptomycin.

### 2.3. BiFC Assays

Cells were seeded in 48-, 24-, or 12-well plates and grown to at least 50% confluence before being transfected with the appropriate amount of each vector using Lipofectamine 2000 (Invitrogen, Carlsbad, CA, USA). A ratio of 1 µg DNA:3 µL Lipofectamine was used for all the experiments. Efficiency was assessed by co-transfection with a pmRFP-TMD (a gift from Dr. José Alberto Carrodeguas, University of Zaragoza) or pAL2-Myc-mRFP vector. Transfections were carried out in the presence of 30 µM Z-VAD-fmk (Bachem) to maintain cell integrity. Transfected cells were cultured at 37 °C for 24 h, and then, Venus (BiFC) and mRFP signals in cells were quantified in a FACSCalibur flow cytometer. A gating analysis based on mRFP fluorescence was performed to exclude non-transfected cells. The mean fluorescence intensities of the BiFC complexes were normalized to the mean fluorescence intensity of mRFP (Supplementary Figure S2). At least 10,000 cells were analyzed in each experiment. In some experiments, ABT-199 (Selleckchem, Houston, TX, USA), A-1155463 (MedChemExpress, Sollentuna, Sweden), or S63845 (Selleckchem) were added 1 h after transfection.

For multicolor experiments, cells were simultaneously transfected with three pBiFC vectors coding for CN-, VN-, and CC-fusions and a vector expressing mRFP. Appropriate controls were performed by transfecting cells with two vectors expressing CN- and CC-fusions or VN- and CC-fusions together with the vector containing mRFP cDNA. Cerulean and Venus fluorescences in mRFP-positive cells were determined 24 h after transfection in a FACSAria cytometer equipped with 405 nm, 488 nm, and 635 nm lasers.

### 2.4. Cell Death Analysis

Cell death was analyzed by determining phosphatidylserine (PS) exposure. Cells were incubated for 15 min at room temperature in 100 µL of annexin-binding buffer (2.5 mM NaCl, 140 mM CaCl_2_, 10 mM Hepes/NaOH, pH 7.4) containing 2 µL of annexin V-DY634, prepared as previously described [27]. Cells were diluted to 1 mL with annexin-binding buffer prior to flow cytometry analysis.

### 2.5. CRISPR/Cas9

A MiaPaca2 Bak KO cell line was generated through CRISPR-Cas9 editing. DNA oligos for the sgRNA targeting site (up: CACCGGTCCTCCCAGGCAGGAGTG; down: AAACCACTCCTGGCCTGGGGAGGACC) were annealed and ligated into *Bbs*I digested pSpCas9(BB)-2A-Puro (PX459) V2.0 plasmid (Addgene). Cells were transfected with Lipofectamine 2000 (Invitrogen) and selected with puromycin. Knock-down of Bak protein was verified by Western Blot.

### 2.6. Western Blot Analysis

To determine the expression level of the fusion proteins, SDS-PAGE and Western Blot analysis of cytoplasmic cell lysates was performed. Cell extracts were prepared in lysis buffer (50 mM Tris/HCl pH 7.6, 150 mM NaCl, 10% (*v*/*v*) glycerol, 1 mM Na_3_VO_4_, 10 mM Na_4_P_2_O_7_, 10 mM NaF, 1 mM EDTA, 10 µg/mL leupeptin, 1 mM PMSF, and 1% (*v*/*v*) Triton X-100) and protein content was determined by BCA assay (Thermo Scientific, Carlsbad, CA, USA). After SDS-PAGE electrophoresis, proteins were transferred to nitrocellulose membranes. Proteins were immunodetected by using appropriate primary antibodies and peroxidase-labeled secondary antibodies (Sigma–Aldrich, St Louis, MO, USA). Bands were visualized with Pierce ECL or ECL Plus Western Blotting Substrate (Thermo Scientific). Specific antibodies against the following proteins were used as follows: Bcl-X_L_ (Cell Signaling Technology, Danvers, MA, USA); Bcl-2 (Abcam, Cambridge, UK); Mcl-1 (Santa Cruz Biotechnology, Dallas, TX, USA); Bim (Merck Millipore, Darmstadt, Germany); Puma (Abcam); Noxa (Santa Cruz Biotechnology); Bax (BD Biosciences); and Bak (Santa Cruz Biotechnology and Millipore, Darmstadt, Germany). The protein-loading control was achieved by membrane reprobing with an anti-β-actin or anti-α-tubulin antibody (Sigma–Aldrich).

### 2.7. Fluorescence Microscopy

Mitochondrial localization of complexes formed by VN- and VC-fusions was analyzed by confocal microscopy. HeLa cells were seeded in coverslips and co-transfected, as previously indicated, with pBiFC vectors expressing fusions of the indicated proteins with VN and VC fragments and pmRFP-TMD, a vector expressing a mitochondrial-targeted mRFP. Following 24 h after transfection, nuclei were stained with Hoechst 33342 (2 µg/mL), cells fixed with 4% paraformaldehyde (PFA) for 15 min at 4 °C and mounted on Fluoromount-G. Images were collected in a sequential mode in a FluoView FV10i (Olympus) confocal microscope with a 60× oil immersion lens, a line average of 4, and a format of 1024 × 1024 pixels. The confocal pinhole was 1 Airy unit. Images were exported without image manipulation from FV10-ASW viewer software (V2.0, Olympus NDT Inc., Waltham, MA, USA) to generate the figures. For fluorescence microscopy, cells were grown on coverslips and transfected as previously described. Transfected cells were stained with 100 nM MitoTracker Red (CMXRos, Invitrogen) 24 h after transfection, washed with PBS, and fixed in 4% PFA at room temperature for 20 min. Finally, coverslips containing fixed cells were washed and mounted in Fluoromount-G (Southern Biotechnology, Birmingham, AL, USA). Images were collected using an Olympus IX81 fluorescence microscope. Images were exported without image manipulation to generate the figures.

### 2.8. Statistical Analysis

All statistical analyses were performed via GraphPad Prism (version 9, GraphPad Software, LLC, San Diego, CA, USA).

## 3. Results

### 3.1. The Interactome of the Bcl-2 Family in Living Cells

**Figure 1:**
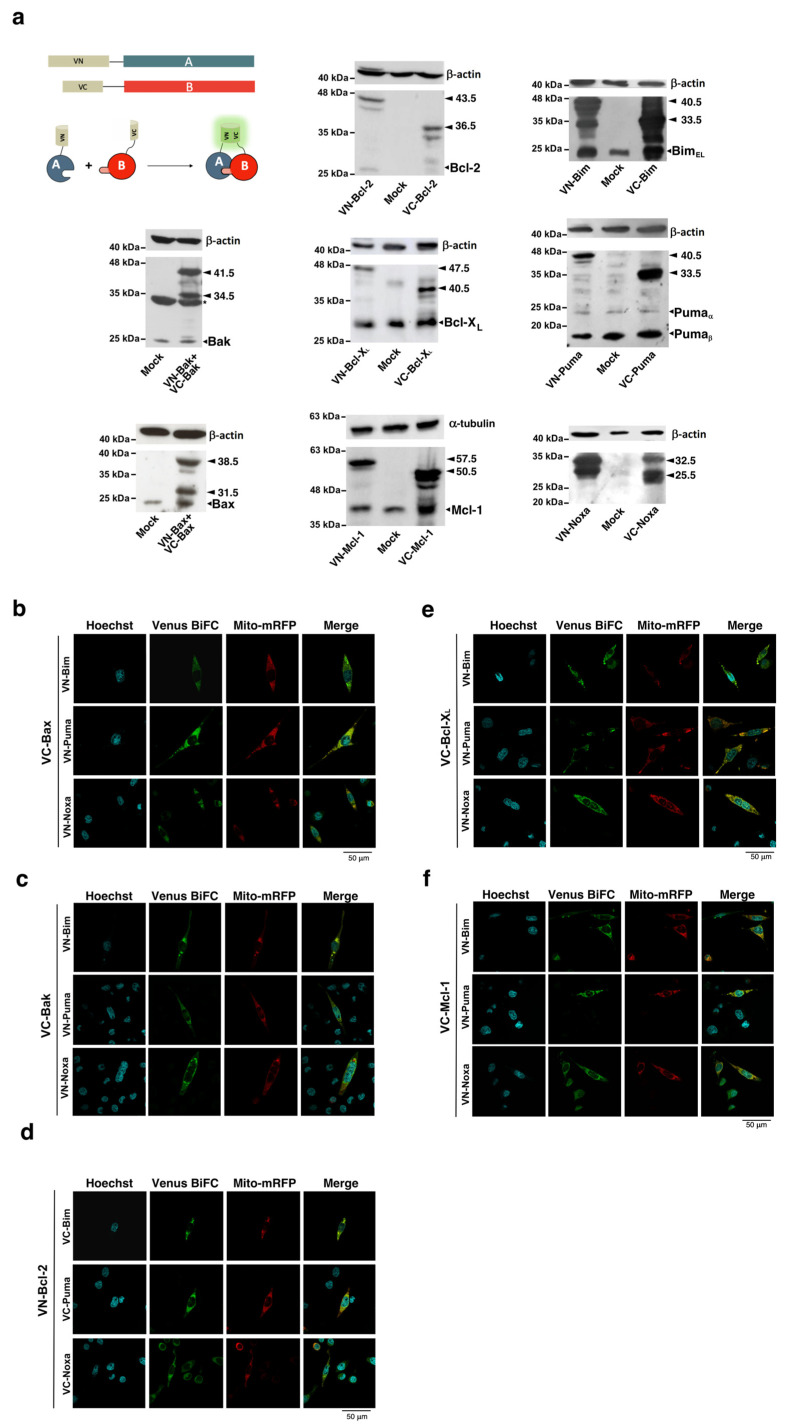
(**a**) Protein fusions used to study interactions between proteins of the Bcl-2 family. Each protein was fused to the VN or VC fragment of Venus protein. After transfection, interaction of VN-A and VC-B allows for complementation of Venus fragments, and fluorescence can be detected. Expression of the BiFC fusions in HeLa cells (VN or VC fragments fused to Bcl-2, Bcl-X_L_, Mcl-1, Bim, Puma, Noxa, Bax and Bak) was analyzed by Western Blot. Arrows indicate the bands of fusions, being the MW of VN-linker 17.5 kDa and VC-linker 10.5 kDa. Small arrows indicate bands corresponding to endogenous proteins. The asterisk denotes a non-specific band. (**b**–**f**) Complexes detected by BiFC show mitochondrial localization. HeLa cells were seeded in coverslips and co-transfected with vectors expressing fusions of the indicated proteins with VN and VC fragments and a vector expressing a mitochondrial-targeted mRFP. Following 24 h after transfection, nuclei were stained with Hoechst 33342 (2 µg/mL), cells fixed with 4% PFA for 15 min at 4 °C, and mounted on Fluoromount-G. Images were collected in sequential mode in a FluoView FV10i (Olympus) confocal microscope, as detailed in the Materials and Methods section.

Control of cell fate upon cell damage depends on the interaction network among proteins of the Bcl-2 family. The balance between pro- and antiapoptotic members and the interactions that these proteins establish determine whether mitochondrial outer membrane permeabilization, the point of no return in the intrinsic pathway, will occur. Bimolecular Fluorescence Complementation can be advantageous compared to other techniques, such as immunoprecipitation, for revealing labile or transient interactions that could be essential in the mode of action of Bcl-2 proteins [28]. Although the fusion of Venus halves to the C-terminus of some proteins of the Bcl-2 family does not preclude correct mitochondrial localization [11], we have generated N-terminal fusions for all the proteins and verified the correct expressions of all the constructs (Figure 1a). Furthermore, the topology of the protein fusions must be optimized for BiFC assays to avoid false negative results [29] that could be caused by interference of the Venus fragments with the interaction surface or by steric hindrance preventing the fluorescent fragment from approaching the right orientation. Thus, to exclude any interference of the Venus fragments in the interaction between proteins or spatial limitations in the complementation of Venus fragments, we generated fusions of both VN and VC fragments of Venus at the N-terminus of all the proteins analyzed, and appropriate pairs were co-transfected in HeLa cells. Equivalent expression of the fusions was confirmed through Western Blot (Figure 1a), excluding the possibility of reduced Venus fluorescence, which was due to low expression of the fusions. For each protein pair, the most favorable configuration of BiFC fusions was used in further experiments. Punctate distribution of BiFC complexes and colocalization with a mitochondria-targeted mRFP confirmed mitochondrial localization (Figure 1b–f).

**Figure 2:**
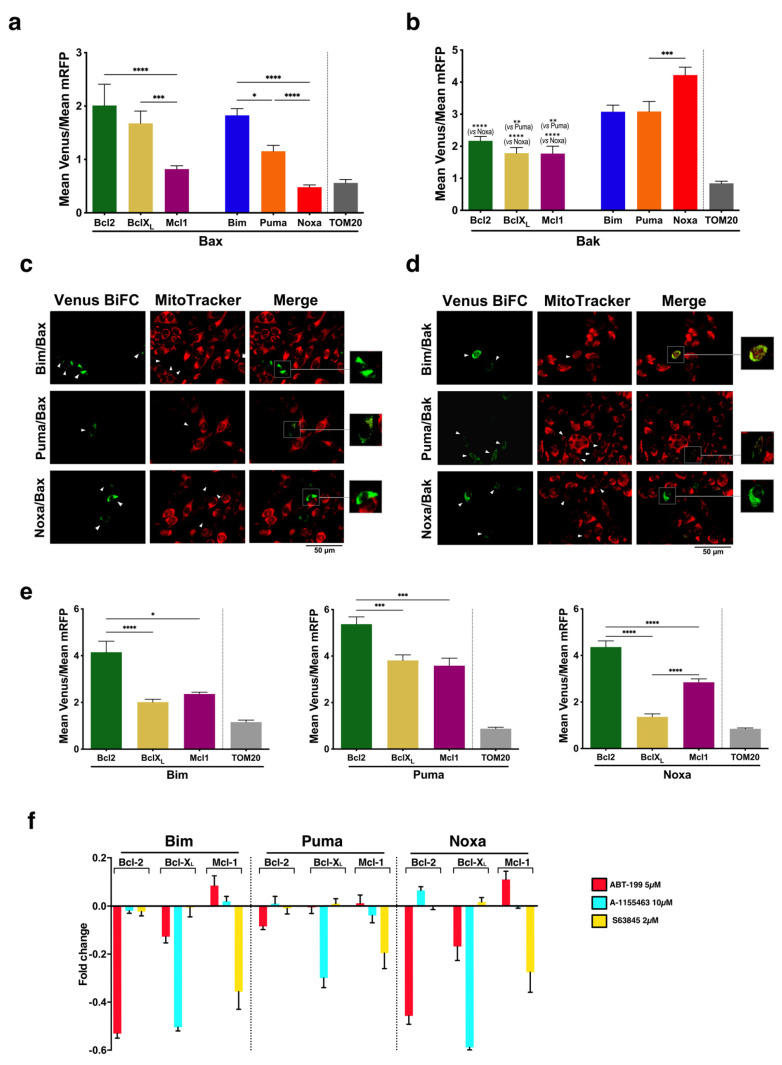
Analysis of Bax and Bak interactions in living cells. (**a**,**b**) Quantification of the interactions of Bax (**a**) and Bak (**b**) with antiapoptotic and BH3-only proteins. HeLa cells were transfected with appropriate pairs of BiFC vectors expressing protein fusions with the VC or VN fragments of Venus: VC-Bax and VC-Bak were co-transfected with VN-Bcl-2, VN-Bcl-X_L_, VN-Bim, VN-Puma, and VN-Noxa; VN-Bax and VN-Bak were co-transfected with VC-Mcl-1. Corresponding TOM20 fusions were used as control. The pmRFP-TMD vector was included in the transfections to allow for gating of transfected cells and normalization of fluorescence intensities. Venus and mRFP fluorescence were analyzed by flow cytometry 24 h after transfection. The Venus/mRFP MFI ratios are represented for each pair of proteins. Results are mean ± SEM of 9 (Mcl-1, Bcl-2, and Bim), 9 (Bcl-X_L_), 11 (Puma and Noxa), and 4 (TOM20) independent experiments. (**c**,**d**) Visualization of BiFC complexes and mitochondria stained with MitoTracker Red. Cells were seeded in coverslips and transfected 24 h later with corresponding BiFC vectors, as indicated in A and B. Following 24 h after transfection, cells were stained with 100 nM MitoTracker Red for 15 min at 37 °C. Then, cells were washed and fixed with 4% PFA. Coverslips were mounted with Fluoromount-G and observed in a fluorescence microscope. (**e**) Association of Bim, Puma and Noxa with antiapoptotic proteins was quantified as indicated in (**a**,**b**). Cells were transfected with pBiFC vectors for expression of appropriate VN- and VC-fusions (VN-Bcl-2 was co-transfected with VC-Bim, VC-Puma or VC-Noxa; VC-Bcl-X_L_ and VC-Mcl-1 were combined with VN-Bim, VN-Puma, or VN-Noxa) together with the pmRFP-TMD. The Venus/mRFP ratio in mRFP-positive cells was determined by flow cytometry. Results are mean ± SEM of 8 independent experiments. (**f**) BH3-mimetics specifically disrupt interactions between antiapoptotic and BH3-only proteins. Cells were transfected as indicated in (**e**), and 1 h after transfection, each ABT-199, A-1155463, or S63483 was added at the indicated concentrations. Venus and mRFP fluorescence were analyzed by flow cytometry 24 h after transfection. Changes in the Venus/mRFP ratios for each inhibitor are represented. Results are mean ± SEM of 3 independent experiments. * *p* < 0.05, ** *p* < 0.01, *** *p* < 0.005, **** *p* < 0.001 (one-way ANOVA followed by Tukey’s multiple comparison test).

For weak interactions, the intensity of detected fluorescence has been proposed to be proportional to the strength of the interaction [30]. On this premise, we analyzed the interactions between various pairs of Bcl-2 proteins in order to delineate a “Bcl-2 interactome” in living cells. HeLa cells were transfected with VN/VC-fusion pairs, and the fluorescence intensity was quantified by means of flow cytometry (Figure 2, Supplementary Figure S1).

Cells were co-transfected with a vector containing the cDNA of mRFP to allow for the gating of transfected cells (which ranged from 30% to 50% in every experiment), and Venus/mRFP fluorescence was calculated (Supplementary Figure S1). We first compared the interactions of the multidomain proteins Bak and Bax with antiapoptotic proteins or BH3-only proteins (Figure 2a,b). Mitochondrial Tom20 protein was used as a negative control, as previously reported [31]. Our results showed that both Bax and Bak interacted with Bcl-2, Bcl-X_L,_ and Mcl-1, although the Mcl-1/Bax interaction seemed to be weaker, according to a lower Venus intensity detected for the Mcl-1/Bax pair (Figure 2a). To exclude that the differences observed in Venus complementation could be due to differential expression of the constructs, we performed WB analysis using anti-Flag and anti-HA antibodies, which allowed the detection of different constructs in the same membrane (Supplementary Figure S2). On the other hand, we observed differences in the ability of Bax and Bak to bind BH3-only proteins. Venus complementation intensities suggested that Bak binds more easily to BH3-only proteins than Bax (Figure 2a,b). Among BH3-only proteins analyzed, Bim and Puma seemed to be the most probable activators of Bax. In contrast, Bak/Noxa complexes gave the highest fluorescence intensity for Bak. Fluorescence microscopy showed that complexes between Bax, Bak, and BH3-only proteins displayed a punctate pattern (Figure 2c,d), according to the mitochondrial localization detected by confocal microscopy (Figure 1b–f). Moreover, Venus-positive cells displayed reduced MitoTracker Red staining, suggesting loss of mitochondrial transmembrane potential. Interactions of Bim, Puma, and Noxa with antiapoptotic proteins were also evaluated. As shown in Figure 2e, Bim associates with Bcl-2 and, to a lesser extent, with Bcl-X_L_ and Mcl-1. The intensity of Venus fluorescence for the Bim/Bcl-2 pair was similar to that of the Bim/Bak pair. Puma is strongly associated with the three antiapoptotic proteins (Figure 2e), yielding greater intensities than the Puma/Bax and Puma/Bak complexes (Figure 2a,b). Finally, we observed that Noxa could bind to Bcl-2 and Mcl-1, but it showed a very low affinity for Bcl-X_L_.

We also analyzed the effect of specific BH3-mimetic compounds in the interaction of antiapoptotic proteins with Bim, Puma and Noxa. As shown in Figure 2f, ABT-199 significantly reduced the formation of BiFC complexes of Bcl-2 with Bim, Puma, or Noxa, especially Bim and Noxa. In the same way, A-1155463 and S63845 reduced the interaction of Bim, Puma, and Noxa with Bcl-X_L_ or Mcl-1, respectively.

**Figure 3:**
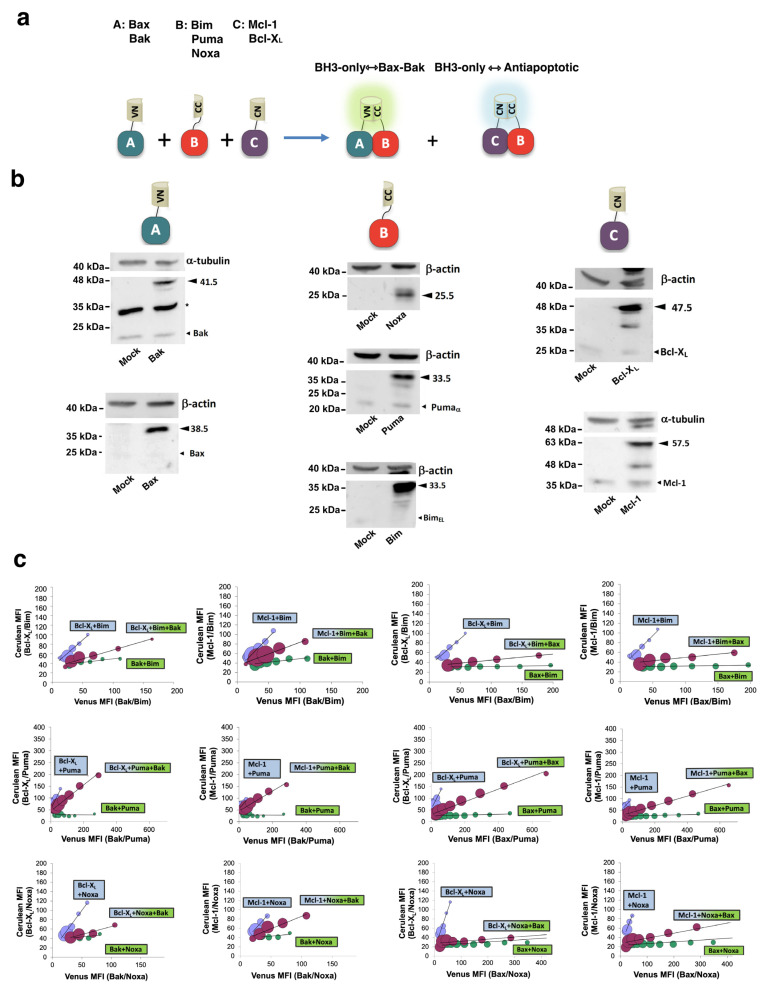
Multicolor BiFC analysis of interactions between BH3-only and antiapoptotic or Bax/Bak proteins. (**a**) Experimental basis of the multicolor assays. Fusions of proteins in each subset with the amino or carboxy fragments of Cerulean (CN and CC, respectively) and Venus (VN) proteins were constructed, as described in the Materials and Methods section. The VN and CN fragments can complement the CC fragment, yielding complete proteins with distinct spectral properties. (**b**) Expression of the CC-, VN-, and CN-fusions was verified through Western Blot. Arrows indicate the bands of fusions, being the MW of CN- and VN-linker 17.5 kDa and CC-linker 10.5 kDa. Small arrows indicate bands corresponding to endogenous proteins. The asterisk denotes a non-specific band. (**c**) HeLa cells were transfected with vectors expressing the fusions indicated in boxes, together with the pAL2-Myc-mRFP vector. Cerulean and Venus fluorescence signals were analyzed in mRFP-positive cells by flow cytometry. FL-9 (cerulean)/FL-1 (Venus) histograms were analyzed using Weasel software. Each histogram was divided into 15 sections in the FL-1 dimension. Mean Venus and Cerulean fluorescences in each section were recorded, represented as an XY graphic, and adjusted to a linear function using GraphPad Prism software. Circle diameter is proportional to cell density in the corresponding section of the histogram. A representative graphic of two independent experiments for each assay is represented.

To gain further insight into the complex interactions network that regulates the activity of Bcl-2 proteins, we performed multicolor bimolecular fluorescence complementation assays (Figure 3a). This technique is based on the fusion of fragments of spectrally distinct fluorescent proteins, allowing us to analyze the competition between interaction partners [32].

**Figure 4:**
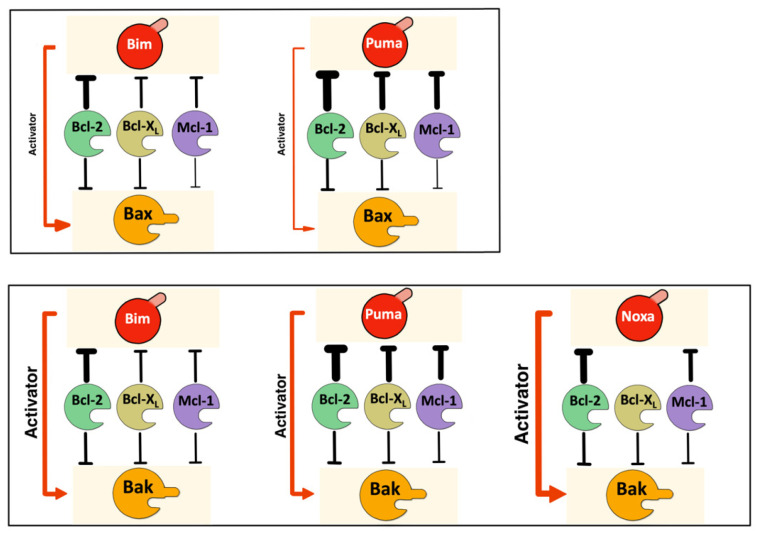
Interactome of Bax/Bak, antiapoptotic, and BH3-only proteins, according to BiFC, results in living cells.

Similar levels of expression of the fusions with Venus and Cerulean fragments were verified by Western Blot (Figure 3b). We analyzed the relative affinity of Bim, Puma, and Noxa to the antiapoptotic Bcl-X_L_ and Mcl-1 proteins versus the multidomain proapoptotic members of the family (Figure 3c). Dot-plot diagrams recording Venus and Cerulean intensities were converted into density graphics, and linear adjustment was performed (Supplementary Figure S3). Cerulean and Venus fluorescence signals were analyzed in mRFP-positive cells by flow cytometry. FL-9 (cerulean)/FL-1 (Venus) histograms were divided into 15 sections in the FL-1 dimension. Mean Venus and Cerulean fluorescences in each section were recorded, represented as an XY graphic, and adjusted to a linear function using GraphPad Prism software. Cells that were positive for mRFP only were excluded from the analysis. Each point represents the Venus (X-axis) and Cerulean fluorescence (Y-axis) in these regions. The number of events in each section was represented by the diameter of the circles. These competition assays suggested that Bax/Bim interaction was favored versus Mcl-1/Bim or Bcl-X_L_/Bim. Furthermore, Bcl-X_L_ was relatively inefficient in avoiding the Bim/Bak association, while Mcl-1 was able to displace an important fraction of Bim, preventing its binding to Bak, as indicated by the slopes of cell populations in dot-plot diagrams corresponding to triple transfection with CC-, CN-, and VN-fusions (Figure 3c). In contrast, Puma seemed to associate preferentially with antiapoptotic proteins when competing with Bak (Figure 3c). The Puma/Bax association was also partially reduced by both antiapoptotic proteins. Finally, Venus/Cerulean fluorescence representation indicated that Mcl-1 could compete with Bax and Bak for Noxa (Figure 3c). However, Venus fluorescence was predominant in the Bcl-X_L_/Noxa/Bax and Bcl-X_L_/Noxa/Bak combinations, according to the low binding of Bcl-X_L_ to Noxa observed in single-color BiFC assays compared to Mcl-1/Noxa and Bcl-2/Noxa. Although Bcl-X_L_/Noxa and Bax/Noxa complexes displayed similar fluorescence ratios in cells transfected only with two fusions (Figure 2a,e), multicolor experiments suggested that Noxa bound preferentially to Bax when both Bcl-X_L_ and Bax fusions were expressed simultaneously. Considering both the single-color BiFC experiments and the multicolor competition assays, an interactome map for the Bcl-2 family can be outlined (Figure 4).

### 3.2. Involvement of the α1 Helix and the BH3 Domain in the Interaction of Bax and Bak with BH3-Only and Antiapoptotic Proteins

**Figure 5:**
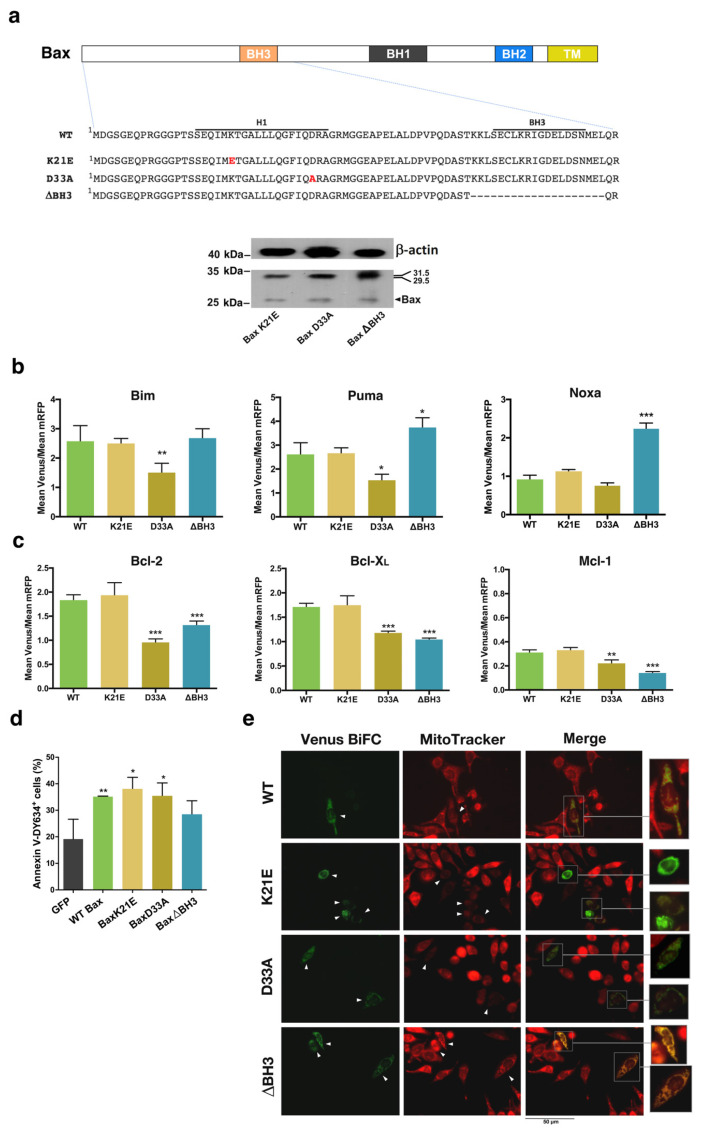
Role of α1 helix and BH3 domain in the interactions of Bax protein with BH3-only and antiapoptotic members of the Bcl-2 family. (**a**) Wild-type and mutated VN/VC-Bax fusions were used for BiFC. Expression of the mutants was confirmed by means of Western Blot. Theoretical MW of the fusions is indicated on the right side of each gel (VN fragment 17.5 KDa; VC fragment 10.5 KDa). (**b**) HeLa cells were transfected with vectors expressing Bim, Puma, or Noxa fused to the VN Venus fragment together with vectors expressing the corresponding Bax wild-type or mutated fusions with the VC Venus fragment and the pmRFP-TMD vector. Mean Venus/mRFP fluorescence intensity ratios for each pair are indicated. Results are the mean ± SEM of 4 (Bim, Puma) or 6 (Noxa) independent experiments. (**c**) HeLa cells were transfected with vectors expressing Bcl-2, Bcl-X_L,_ or Mcl-1 fusions with the VN (Bcl-2 and Bcl-X_L_) or the VC (Mcl-1) Venus fragment together with vectors expressing the corresponding Bax wild-type or mutated fusions and the pmRFP-TMD vector for mRFP expression. Mean Venus/mRFP fluorescence intensity ratios for each pair are indicated. Results are the mean ± SEM of 4 (Bcl-2 and Bcl-X_L_) or 6 (Mcl-1) independent experiments. * *p* < 0.05, ** *p* < 0.01, *** *p* < 0.005. One-way ANOVA followed by Tukey’s multiple comparison test. (**d**) HCT116 Bax^−/−^ cells were transfected with pBabe vectors expressing WT or mutated Bax protein. A vector expressing GFP was used as a control for unspecific cell death. The percentage of apoptotic cells was analyzed 48 h after transfection by Annexin V-DY634 binding and flow cytometry. Results are mean ± SEM of 3 independent experiments. * *p* < 0.05, ** *p* < 0.01. One-way ANOVA followed by Tukey’s multiple comparison test. (**e**) Cells were seeded in coverslips and transfected 24 h later with corresponding BiFC vectors for expression of VN-Bcl-X_L_ and the indicated VC fusions. Following 24 h after transfection, cells were stained with 100 nM MitoTracker Red for 15 min at 37 °C. Then, cells were washed and fixed with 4% PFA. Coverslips were mounted with Fluoromount-G and observed in a fluorescence microscope.

**Figure 6:**
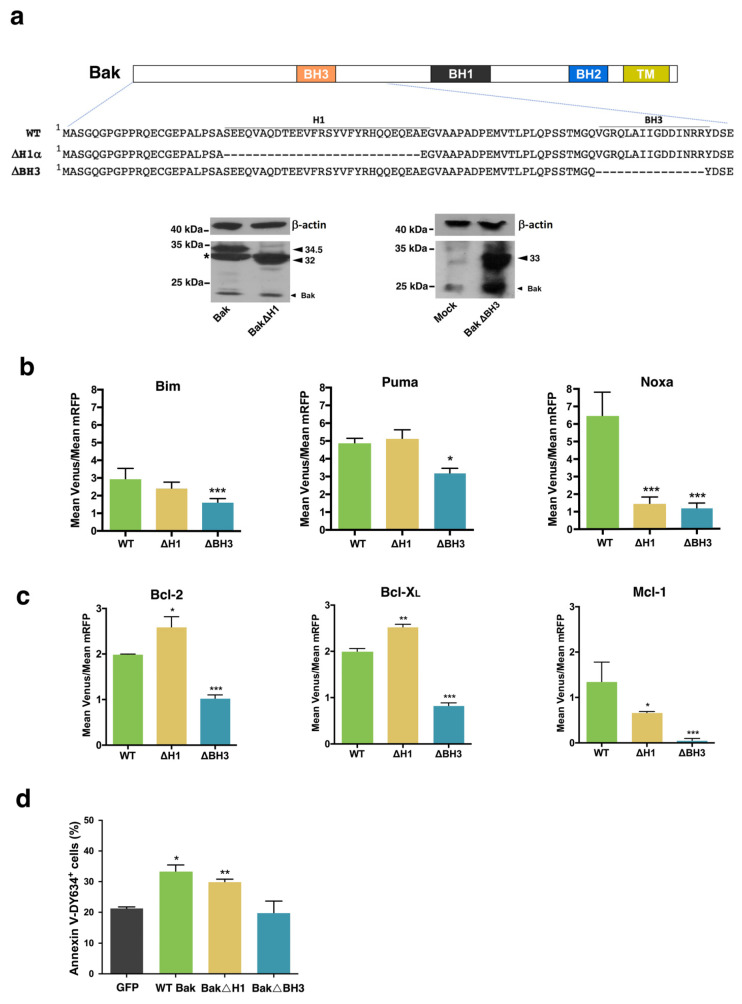
Role of α1 helix and BH3 domain in the interactions of Bak protein with BH3-only and antiapoptotic members of the Bcl-2 family. (**a**) Wild-type and mutated VN/VC-Bak fusions were used for BiFC. Expression of mutants was confirmed by Western Blot. For detection of the ΔBH3 mutant, the anti-Bak (NT) antibody from Upstate was used, while an anti-Bak (G-23) antibody from Santa Cruz Biotechnology allowed for detection of WT, ΔH1α, or H164A Bak variants. * Non-specific band. (**b**) HeLa cells were transfected with vectors expressing Bim, Puma, or Noxa fused to the VN Venus fragment together with vectors expressing the corresponding Bak wild-type or mutated fusions with the VC Venus fragment and the pmRFP-TMD vector. Mean Venus/mRFP fluorescence intensity ratios for each pair are indicated. Results are the mean ± SEM of 4 independent experiments. (**c**) Hela cells were transfected with vectors expressing Bcl-2, Bcl-X_L_ (VN), or Mcl-1 (VC) fusions with a Venus fragment together with vectors expressing the corresponding Bak wild-type or mutated fusions and the pmRFP-TMD vector. Mean Venus/mRFP fluorescence intensity ratios for each pair are indicated. Results are the mean ± SEM of 5 independent experiments. * *p* < 0.05, ** *p* < 0.01, *** *p* < 0.005. One-way ANOVA followed by Tukey’s multiple comparison test. (**d**) MiaPaca2 Bak^−/−^ cells were transfected with pBabe vectors expressing wild-type or mutated Bak protein. A vector expressing GFP was used as a control for unspecific cell death. The percentage of apoptotic cells was analyzed 48 h after transfection by Annexin V-DY634 binding and flow cytometry. Results are mean ± SEM of 3 independent experiments. * *p* < 0.05, ** *p* < 0.01. One-way ANOVA followed by Tukey’s multiple comparison test.

The rear region of Bax and Bak has been proposed to be the binding site for activator BH3-only proteins [14]. Thus, we studied the interaction of BH3-only and antiapoptotic proteins with Bax and Bak mutants in the α1 helix and BH3 regions (Figure 5 and Figure 6). Expressions of the fusions containing mutant Bax or Bak were verified by Western Blot analysis (Figure 5a and Figure 6a). As shown in Figure 5b, Bax D33A mutants displayed a decreased affinity for the three BH3-only proteins analyzed, suggesting that negative charges in this region of Bax could be involved in the binding of activator BH3-only proteins. On the contrary, the K21E mutation did not alter the interaction of Bax with Bim, Puma, or Noxa. However, BH3 deletion mutants of Bax even increased their association with Puma and Noxa. These results point to the α1 helix as the binding site for BH3-only proteins, as previously proposed [14], and confirm the critical involvement of D33 in the activation of Bax.

We also explored the involvement of the α1 helix and the BH3 domain in the interaction of Bax with antiapoptotic proteins. As shown in Figure 5c, both the D33A mutation and deletion of the BH3 domain significantly reduced the interaction between Bax and antiapoptotic proteins. These results suggest that both interfaces could be binding sites for antiapoptotic proteins. However, both the K21E and the D33A mutants induced apoptosis at the same level as the wild-type protein (Figure 5d), and fluorescence microscopy also suggested that mitochondrial transmembrane potential was dissipated after transfection with Bcl-X_L_ combined with Bax K21E or Bax D33A fusions (Figure 5e). In contrast, the ΔBH3 mutant displayed reduced proapoptotic activity (Figure 5d). Furthermore, Venus-positive cells, after transfection with the Bcl-X_L_/ΔBH3 pair mutant, seemingly kept high mitochondrial transmembrane potential (Figure 5e), indicating that the BH3 domain is essential for mitochondrial permeabilization.

Deletion of the α1 helix of Bak (Figure 6a) did not affect the interaction with Bim and Puma (Figure 6b). However, the ΔH1α mutant of Bak exhibited a reduced ability to bind Noxa. On the other hand, our results show that the BH3 domain of Bak is involved in the binding of Bim, Puma, and Noxa. The affinity between Bak and Bim or Puma was decreased when the BH3 domain of the former was deleted, but complexes were still detected and the Venus/mRFP ratio was only slightly reduced, suggesting this mutation affected the affinity of Bak for these BH3-only proteins, probably due to alterations of the canonical groove which includes the BH3 domain. According to accepted models, we observed that ΔBH3 Bak mutants almost lost the ability to interact with antiapoptotic proteins. However, ΔH1α mutants yielded higher Venus intensities when combined with Bcl-2 or Bcl-X_L_ fusions (Figure 6c). This finding could be explained by the constitutive activation of the truncated protein, as reported for Bax isoforms lacking the N-terminus [33]. Surprisingly, the ΔH1α Bak mutant also showed a reduced interaction with Mcl-1. This could indicate that the interaction between Mcl-1 and Bak could occur through different surfaces other than the BH3 domain. In agreement with these observations, ΔH1α Bak, but not ΔBH3 Bak, induced apoptosis in Bak KO cells generated by CRISPR-Cas9 (Figure 6d).

### 3.3. Involvement of the BH3 Domain and the α1–α6 Interface in Dimerization of Bax and Bak

**Figure 7:**
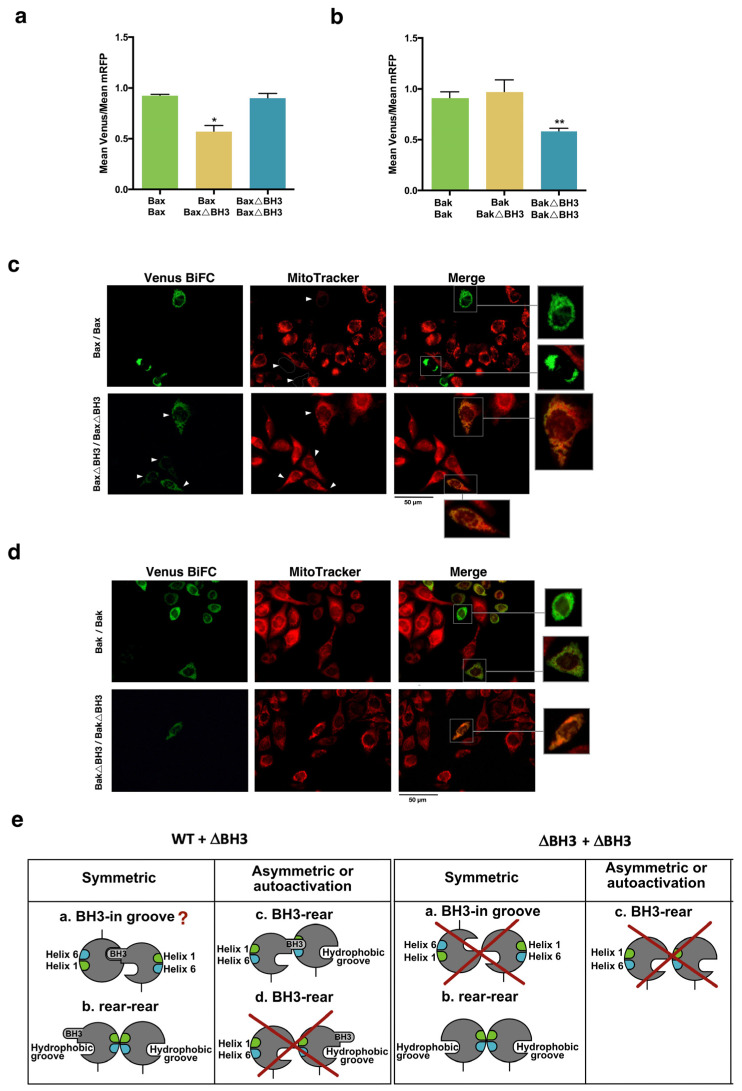
Analysis of the dimerization between Bax and Bak WT and ΔBH3 mutants. Cells were transfected with vectors expressing wild-type and ΔBH3 Bax (**a**) or Bak (**b**) fused to VC or VN fragments, as indicated, together with pmRFP-TMD vector. Venus/mRFP ratios were analyzed by flow cytometry. Results are mean ± SEM of 10 (Bax) or 6 (Bak) independent experiments. * *p* < 0.05, ** *p* < 0.01. One-way ANOVA followed by Tukey’s multiple comparison test. (**c**,**d**) ΔBH3 Bax and Bak mutants show reduced proapoptotic activity. Hela cells were transfected with vectors expressing VN-Bax/VC-Bax and VN-BaxΔBH3/VC-BaxΔBH3 (**c**) or VN-Bak/VC-Bak and VN-BakΔBH3/VC-BakΔBH3 (**d**) and 24 h later cells were stained with 100 nM MitoTracker Red. Venus fluorescence and MitoTracker Red were visualized in a fluorescence microscope. Arrowheads point to Venus-positive cells (**e**) Dimerization between WT and ΔBH3 mutants according to the symmetric and asymmetric models. Possible dimerization interfaces for each model are depicted. Crosses denote protein interactions not allowed due to mutations. Interrogation marks indicate possible interactions that could still occur depending on the surfaces involved in dimerization and oligomerization of Bax and Bak.

We next explored the dimerization process using fusions of Venus fragments with wild-type or mutants of both Bax and Bak proteins. In dimerization experiments, the Venus/mRFP ratios were lower than that measured for most of the heterodimers (Figure 1, Figure 2, Figure 3, Figure 4 and Figure 5), probably due to the dimerization of fusions with non-complementary Venus fragments (Supplementary Figure S4). Nevertheless, we observed some significant changes when mutants in the α1 helix, the α6 helix, and the ΔBH3 variants were used to explore the interfaces involved in the homodimerization of both proteins. Deletion of the BH3 domain from one of the Bax fusions reduced dimerization leading to Venus complementation (Figure 7a).

In this case, the reduction in Venus complementation could be caused by destabilization of the BH3-in-groove interaction when the BH3 domain of one of the Bax fusions is deleted. Alternatively, this reduction of fluorescence could probably be due to the dimerization of the wild-type proteins fused to the same Venus fragment that can associate in a BH3-in-groove configuration without yielding Venus complementation (Supplementary Figure S4). Further studies would be required to clarify this point. However, when both fusions were constructed with ΔBH3 mutants, Venus fluorescence intensity was similar to that of wild-type Bax dimers (Figure 7a), indicating that dimerization can occur by the interaction of other domains, such as the rear interface (Figure 7e, symmetric “b”), but would not be compatible with an asymmetric model. In this case, the association of non-fluorescent dimers of fusions with the same Venus fragment would not be favored since both fusions lack the BH3 domain. Importantly, although ΔBH3 mutants could dimerize, these fusions were unable to induce mitochondrial dysfunction, as suggested by the detection of transmembrane potential with MitoTracker Red (Figure 7c). Deletion of the BH3 domain only caused a decrease in dimer formation when both fusions contained truncated Bak (Figure 7b). The fact that the WT/ΔBH3 pair yielded Venus complementation at the same level as WT/WT transfection could indicate that the BH3 domain of the WT fusion can still bind to the partial hydrophobic groove in the ΔBH3 fusion (Figure 7e, symmetric “a”). On the other hand, when cells were transfected with BakΔBH3 fusions, we did not observe a loss of mitochondrial transmembrane potential (Figure 7d), confirming that the BakΔBH3 fusion has lost proapoptotic functionality.

**Figure 8:**
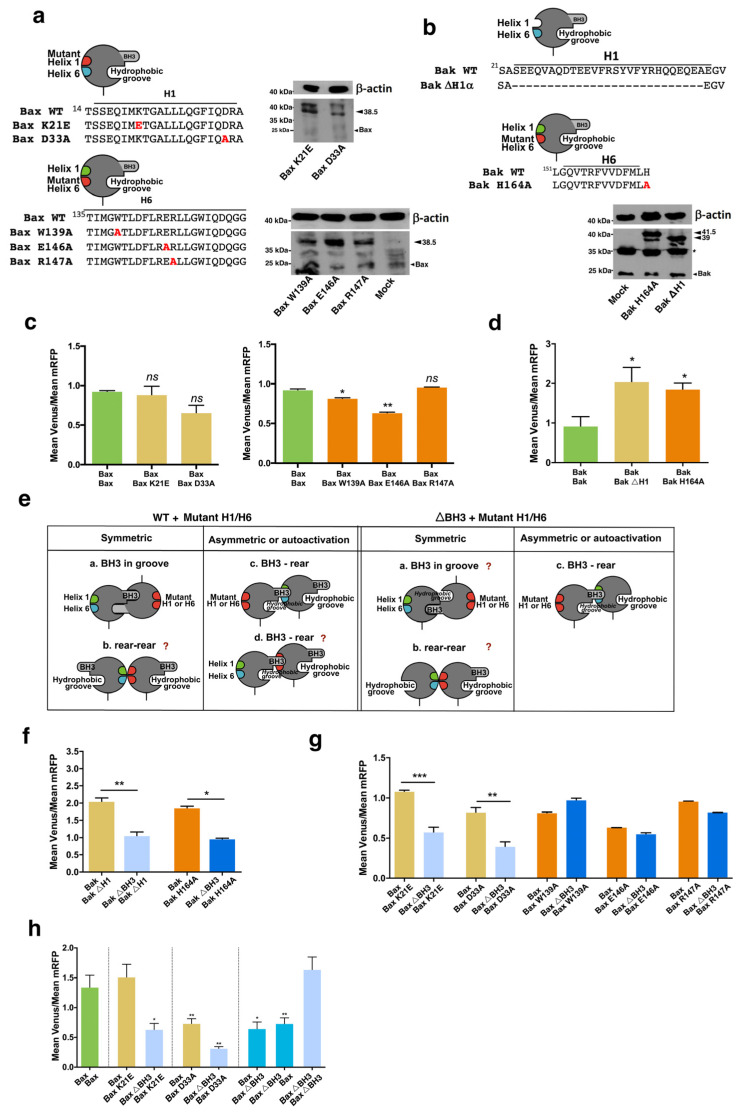
Involvement of α1 and α6 helices in Bax and Bak oligomerization. pBiFC vectors with Bax (**a**) or Bak (**b**) mutants in the helix α1 or α6 were constructed, and their expression was verified by Western Blot. * Non-specific band. (**c**) Cells were transfected with vectors for the expression of wild-type Bax α1 or α6 mutants indicated, fused to VC or VN fragments and the pmRFP-TMD vector. Venus/mRFP ratio for each pair of fusions is represented. Results are mean ± SEM of 6 (W139A, E146A, and R147A) or 8 (WT and K21E and D33A) * *p* < 0.05, ** *p* < 0.01 One-way ANOVA followed by Tukey’s multiple comparison test. (**d**) Cells were transfected with vectors for the expression of wild-type Bak, and α1 or α6 helix mutants indicated, fused to VC or VN fragments. The pmRFP-TMD vector was included in the transfection. Venus/mRFP ratio for each pair of fusions is represented. Results are mean ± SEM of 6 (H164A) or 8 (WT and ΔH1α) independent experiments. * *p* < 0.05, One-way ANOVA followed by Tukey’s multiple comparison test. (**e**) Possible interaction interfaces between WT and α1/α6 mutants or ΔBH3 and α1/α6 mutants. (**f**,**g**) Cells were transfected with the indicated pairs of fusions for BiFC, together with the pmRFP-TMD vector. Venus/mRFP mean fluorescence ratio was determined by flow cytometry. Results are mean ± SEM of 6 (**f**) or 3 (**g**) independent experiments. * *p* < 0.05, ** *p* < 0.01, *** *p* < 0.005. Student two-tailed unpaired *t*-test. (**h**) Bax dimerization analysis by BiFC in HCT116 Bax^−/−^ cells. Cells were transfected with vectors for BiFC containing the cDNA of the indicated wild-type or mutant Bax proteins fused to VN/VC Venus fragments, and the pmRFP-TMD vector for expression of mRFP was included for selection of transfected cells and fluorescence normalization. Venus/mRFP ratios were determined by flow cytometry 24 h after transfection. Results are mean ± SEM of 4 independent experiments with two replicates. * *p* < 0.05, ** *p* < 0.01 One-way ANOVA followed by Tukey’s multiple comparison test.

These results suggest that a second interface, apart from the BH3-in-groove, must be involved in Bax and Bak dimerization. Data obtained in studies with recombinant proteins point to the α1:α6 as the second interface involved in Bax and Bak oligomerization [20,21]. Thus, we analyzed the dimerization of Bax and Bak mutants in the α1 or the α6 helix (Figure 8a,b). The K21E Bax mutant was able to associate with wild-type Bax at the same level that the non-mutated protein, but mutation of D33 to alanine slightly reduced the dimerization with wild-type Bax, both in HeLa (Figure 8c) and in HCT116 Bax^−/−^ cells (Figure 8h). Two different Bax mutants in the α6 helix, W139A, and E146A, significantly reduced dimerization with the wild-type fusion (Figure 7c). In the same way, we analyzed the involvement of α1 and α6 helices in the dimerization of Bak. Deletion of the α1 helix or mutation of H164 in the α6 helix did not preclude Bak homodimerization, and we even detected higher levels of fluorescence, suggesting a favored association between wild-type and mutated Bak fusions (Figure 8d). In order to gain further insight into the dimerization of Bax and Bak, we also analyzed the interactions between mutants in the helices α1 or α6 with the ΔBH3 fusions (Figure 8e). Deletion of the BH3 domain of Bax reduced the complexes formed with the K21E and D33A mutants in HeLa and HCT116 Bax^−/−^ cells (Figure 8g,h). Taken together, these results suggest that the α1 helix could act as a binding region for Bax-BH3 domains but would not be critically involved in the assembly of dimers through the rear surface. In contrast, combining α6 helix and ΔBH3 mutants did not suppose a further reduction in the association of fusions (Figure 8g), apart from that caused by α6 mutations in one of the fusions (Figure 8c). Interestingly, we did not observe the reduction in fluorescence that occurred in the WT/ΔBH3 pairs (Figure 7a). This could indicate that some of the possible non-fluorescent pairs that could assemble in these experiments (Supplementary Figure S4a, pairs 2, 3, or 6) would not be favored when the a6 helix is mutated. These findings would fit with a model in which the Bax α6 helix takes part in the second interface in oligomerization, but it is not a receptor domain for the BH3 region of a pre-activated Bax molecule. In contrast, deletion of the BH3 domain of Bak reduced dimerization when combined with α1 or α6 mutants (Figure 8f). Considering that deletion of the helix α1 had no effect on the self-association of Bak monomers, the decrease observed in Figure 8f could reflect a decrease in the formation of symmetric dimers due to the lack of one BH3 domain. Since some degree of Venus fluorescence was still detected in all cases, it is possible that these mutations only partially affected the interaction between monomers or that Bax and Bak could dimerize through alternative surfaces, as proposed by some authors [22,23,24].

## 4. Discussion

Interactions among proteins of the Bcl-2 family can regulate the susceptibility of cells to apoptotic death. Unraveling the Bcl-2 interactome could contribute to the improvement of therapy in pathologies in which the deregulation of cell death occurs. Based on this hypothesis, small compounds targeting the antiapoptotic members of the family by mimicking the BH3 domains have been developed. The first-in-class BH3-mimetic reaching the clinic has been ABT-199 (Venetoclax), a Bcl-2-specific inhibitor recently approved for B-CLL, SLL, and AML. Additionally, S63845, a specific and potent Mcl-1 inhibitor, has been described [34], and a related compound is being evaluated in clinical trials in hematological neoplasia [35]. Since cells of different origins can vary in their dependence on antiapoptotic proteins, precise knowledge of the Bcl-2 interactome could help to predict the response of tumor cells to BH3-mimetics or to design new compounds to activate the intrinsic pathway of apoptosis in cancer cells. Data obtained with recombinant proteins or through immunoprecipitation clearly suggest that interactions among Bcl-2 proteins are more promiscuous than initially thought and probably include features of both the “direct” and the “displacement” models. Recent models, such as the “unified model”, include the notion that some BH3-only proteins can directly activate the multidomain effector proteins and also propose that antiapoptotic proteins can act in two different modes, either blocking activator BH3-only proteins (Mode 1) or effector proteins (Mode 2) [36]. These new models delineate a complex network of interactions that must be finely tuned to control cell fate in response to apoptotic stimuli. On the other hand, these interactions can also be modulated by the membranes in which these proteins are located or translocated during apoptosis, such as proposed by the “embedded together” model.

In order to study interactions among proteins of the Bcl-2 family in a cellular context, we have implemented the BiFC assay to a network of proteins in the Bcl-2 family, including antiapoptotic members (Bcl-2, Bcl-X_L,_ and Mcl-1), BH3-only (Bim, Puma, and Noxa), and the two effector multidomain proteins (Bax and Bak). First, we have compared the interactions between pairs of full-length anti- and proapoptotic proteins, which allowed us to delineate an interaction map of them (Figure 4). The interactions of Bax and Bak with Bcl-2, Bcl-X_L_, Mcl-1, Bim, Puma, and Noxa were studied simultaneously, and cells were co-transfected with an mRFP protein to allow for the normalization of Venus fluorescence intensities. We observed some differences in the ability of Bax and Bak to interact with all the proteins analyzed, suggesting differential affinities among its partners. Our results suggest that Bak can be preferentially activated by direct interaction with BH3-only proteins, but Bax may present a mixed mechanism since it displays a similar affinity for antiapoptotic and BH3-only proteins. Regarding the role of BH3-only proteins as activators, our observations confirm that Bim, but also Puma and Noxa, can bind to Bax and especially to Bak, supporting previous reports [6,7,11,14,37,38,39]. A possible explanation of why these interactions remained elusive in many previous studies could lie in their expected transient nature [7,40]. This difficulty is circumvented by the BiFC technique since the complementation of the Venus fragments is irreversible, which is an advantage for the detection of transient or weak interactions. Classification of Puma as a BH3-only activator protein has been controversial. Although Puma co-immunoprecipitates with Bax, the fact that a ΔC mutant, able to bind Bcl-2 but not Bax, still induces apoptosis has been interpreted as evidence that Puma mainly acts as a sensitizer [41]. The study of interactions by BiFC also suggests that Puma can act in both modes but displays a preferential affinity for antiapoptotic proteins. First of all, single-color BiFC experiments showed that Puma associated better with Bcl-2, Bcl-X_L,_ and Mcl-1, as reflected by the high Venus intensities observed. Likewise, using a multicolor BiFC assay, we have verified that Bcl-X_L_ and Mcl-1 could efficiently compete with Bak and Bax to bind Puma, suggesting again that this BH3-only protein can contribute to apoptosis mainly through blocking antiapoptotic members, according to previous results [11]. In contrast, interactions between Bim or Noxa and Bax/Bak prevailed when competing with Mcl-1 and Bcl-X_L_. These results would also agree with previous works showing that Puma BH3 peptides were less efficient MOMP inducers than Bim, tBid, or even Noxa BH3 peptides [39]. Of note, they observed that Bak induces cytochrome c release only after BH3 triggering, suggesting that it depends on direct activation to induce mitochondrial permeabilization. Interestingly, our present results suggest that full-length Bak in cells is better activated by BH3-only proteins than Bax.

On the other hand, the relative binding of BH3-only proteins to antiapoptotic members of the Bcl-2 family can vary among cell types, as recently reported for Bim in myeloma cell lines [42]. This binding preference could determine the sensitivity of cancer cells to BH3-mimetics. Our present results indicate that Bcl-2 has a high affinity for Bim, Puma, and Noxa. In the case of Noxa, this result is inconsistent with the accepted assumption that Noxa is mainly kept in check by Mcl-1. This apparent discrepancy could be explained if the Bcl-2/Noxa interaction were short-lived, as observed by using a Noxa BH3 peptide in vitro [43]. Importantly, the specificity of the interactions detected via the BiFC technique was demonstrated by inhibition with BH3-mimetic compounds. The association of Bim, Puma, and Noxa with each antiapoptotic protein was significantly reduced in the presence of the corresponding BH3-mimetic. BH3-mimetics only partially reduced the interaction between Puma and the three antiapoptotic proteins, suggesting that Puma could bind to antiapoptotic proteins through regions other than the hydrophobic canonical groove targeted by these compounds. Analysis of the interaction between Bax and Bak with the antiapoptotic proteins revealed no significant differences in the interactions of Bcl-2, Bcl-X_L,_ and Mcl-1 with Bak, despite previous reports suggesting that Bcl-2 is unable to interact with this protein [44]. Conversely, Bak and Bcl-2 have been reported to co-immunoprecipitate in protein extracts from lymphoid cells [45], according to our present results.

Another important question in the direct model of Bax and Bak activation concerns the putative binding site for BH3-only proteins. Two different possibilities have been proposed, the α1/α6 rear pocket and the canonical hydrophobic groove formed by helices α2–α5. We have addressed this question using the BiFC assay with mutants affecting both interfaces. Interestingly, we found that the α1 helix seems to be involved in the association of Bim, Puma, and Noxa to Bax, as suggested by the significant decrease in the formation of complexes when cells were transfected with the D33A Bax fusion protein. However, deletion of the BH3 domain of Bax did not hinder its association with Bim and Puma and even increased binding to Noxa, probably due to conformational changes that favored the insertion of the BH3 domain of Noxa in the rear surface of Bax. Altogether, these results point to the rear surface of Bax as the activation site for BH3-only proteins, according to previous reports [10]. Regarding the residues that could be involved in the BH3-Bax rear pocket interaction, the K21E Bax mutant interacted with Bim, Puma, and Noxa and induced mitochondrial depolarization at the same level as the wild-type protein, while mutation of D33 to alanine, partially reduced the interaction of Bax with BH3-only proteins. These results disagree with a previous work in which K21E Bax mutant partially lost the capacity to interact with a Bim SAHB peptide or induce apoptosis in reconstituted Bax/Bak KO cells [10] and would support the involvement of D33, also proposed to be critical for Puma-mediated activation of Bax [9]. Since the effect of the D33A mutation was only partial, we cannot exclude that other residues or other domains could also contribute to Bax activation by binding of full-length Bim.

Although Bax and Bak are considered to fulfill an equivalent function in the intrinsic pathway, they present some structural differences that could reflect different mechanisms of activation. Leshchiner et al. have reported that photoreactive BH3 helices bind to the hydrophobic groove of liposome-reconstituted full-length Bak and to the α1/α6 pocket of Bax [46]. This difference could be explained by the fact that the α9 helix of inactive cytosolic Bax is buried in the canonical hydrophobic groove, probably hindering the binding of BH3-only activator proteins. However, Bak is constitutively inserted in the outer mitochondrial membrane through its α9 helix, and the hydrophobic groove is probably more accessible for BH3-only proteins. Furthermore, Dai et al. found that mutation of R36 in the α1 helix of Bak did not affect its interaction with either Bim or Noxa, while they could detect the binding of these proteins to the hydrophobic groove [7]. Our results with full-length Bim and Puma in a cellular context recapitulate these previous findings since deletion of the BH3 domain, which is part of the hydrophobic groove, reduces the affinity of Bim and Puma for Bak, while deletion of the H1α does not affect these interactions. However, deletion of either the H1α or the BH3 domain of Bak greatly diminishes its association with Noxa, suggesting that both surfaces could act as binding sites for this protein. The ΔH1α Bak mutant was expressed in cells, and it was able to induce apoptosis at the same level as the wild-type protein, excluding a disruptive effect on the stability or functionality of Bak. Our present results could be reconciled with the report of Dai et al. if residues in the α1 other than the R36 were involved in the interaction with Noxa. Alternatively, we cannot disregard that Noxa could bind to a region in Bak altered in both mutants. The fact that some hydrophobic and aromatic residues in the α1 helix can establish contacts with other amino acids in helices α2, α5, and α6 [19], would support this hypothesis. In this case, the alteration caused by α1 deletion might selectively affect the binding of Noxa to the hydrophobic groove without affecting activation by Bim and Puma.

**Figure 9:**
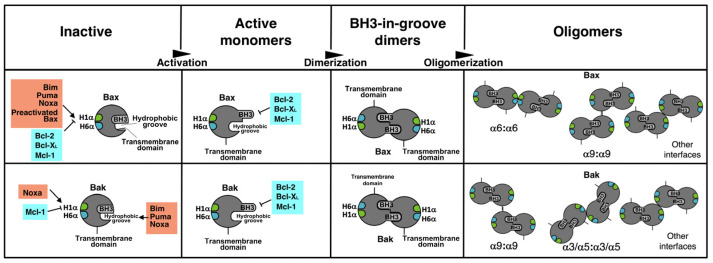
Activation steps for Bax and Bak. Activator BH3-only proteins trigger conformational changes in Bax and Bak, leading to BH3 domain exposure. The trigger site is the α1 helix (H1α) in Bax and the canonical hydrophobic groove (Bim, Puma, Noxa) and the α1 helix (Noxa) in Bak. Preactivated Bax can also activate other Bax molecule by binding to the H1α. Antiapoptotic proteins can block the H1α activation site in Bax and Bak (only Mcl-1). The exposed BH3 domains of activated Bax and Bak can be blocked by antiapoptotic proteins or dimerized through BH3-in-groove interaction. Oligomerization proceeds by BH3-in-groove dimerization, followed by the assembly of dimers in high-order oligomers. The H6α could be involved in the association of Bax dimers, but other interfaces could also mediate its oligomerization. In the case of Bak, interfaces other than the H6α seem to participate in oligomerization.

On the other hand, activated Bax and Bak can be neutralized by the antiapoptotic proteins of the family. The most accepted model for the association of Bax and Bak with antiapoptotic proteins is the “BH3-in-groove”, but also the rear surface of Bax and Bak has been proposed to act as a binding domain for the BH4 domain of antiapoptotic proteins [47,48,49]. The existence of two different sites for Bcl-2/Bax interaction would agree with our findings in living cells since the D33 could be part of the region, which includes the C-terminus of α1, where the BH4 Bcl-2 domain binds to Bax [47]. In this sense, our results with the D33A and ΔBH3 mutants confirm that the interaction of full-length Bcl-2 with Bax could occur at two different sites [47] and further extend this model to Bcl-X_L_ and Mcl-1. Our results also indicate that Bcl-2 and Bcl-X_L_ seem to block Bak exclusively through the canonical BH3-in-groove interaction, in contrast with Bax. Nevertheless, Mcl-1/Bak interaction was also reduced in the H1α mutant, indicating that this protein can block Bak through both interfaces, as observed with Bax (Figure 8). Available data on the binding of antiapoptotic proteins to Bax and Bak have been mainly obtained with truncated forms of these proteins. In the case of Mcl-1, both the N- and the C-terminus were eliminated in two studies aimed at identifying critical residues in the BH3 Bax [50] and Bak [51] domains involved in its interaction with Mcl-1. A possible interaction through the H1α of Bax and the N-terminus of Mcl-1 was not explored, but our results with the full-length protein in living cells suggest this possibility. Taken together, these results suggest that the rear pocket of Bax is a binding site for both antiapoptotic and BH3-only proteins, suggesting that antiapoptotic proteins could prevent in this way direct activation triggered by BH3-only proteins. In the case of Bak, only Mcl-1 and Noxa seem to bind to the rear surface (Figure 9).

We have also applied the BiFC technique to the study of Bax and Bak dimerization. Once again, we have found differences in the way these two proteins self-associate to induce mitochondrial permeabilization. Many reports indicate that once activated, both Bax and Bak form symmetric dimers through the BH3-in-groove association [21,52,53,54,55]. Evidence of the existence of at least two independent surfaces in Bax oligomers in micelles also points to a symmetric model [56]. As an alternative, an asymmetric model was initially proposed by some authors [16], and recent computational work supports that Bak oligomerization proceeds through BH3-rear pocket association to form doughnut-shaped oligomers [38]. Our results in living cells indicate that Bax and Bak monomers can interact, at least, through three interfaces: BH3-in-groove; BH3-rear pocket; and rear pocket-rear pocket. The fact that interaction between two ΔBH3 mutants can be detected by BiFC suggests the existence of at least one interface other than the BH3-in-groove and does not support an asymmetric model of oligomerization. Nonetheless, our results also indicate that the BH3 domain of a Bax molecule can bind to the rear face of another molecule, and the residue D33 participates in this interaction. An alternative explanation would be that this interaction reflects an autoactivation mechanism, previously proposed for Bax [57] and also for Bak [58], rather than an asymmetric oligomerization (Figure 9). Our results with H1α mutants suggest that maybe this autoactivation mechanism could be more important in the case of Bax since deletion of the H1α did not affect the self-association of Bak. Regarding the involvement of α6 helices in dimerization, our results also reveal some differences between Bax and Bak. Mutation of either W139 or E146 in Bax reduced the association of monomers, suggesting that these residues participate in the second dimerization interface, as previously proposed [20,21], while mutation of H164 in Bak did not reduce the level of association. We cannot exclude the involvement of other residues in the helix α6 of Bak in the rear–rear interface, but our results are more compatible with recent reports that propose alternative surfaces for dimer association. In this sense, since some level of dimerization was still detected with all the mutants herein analyzed, it is possible that several interfaces could mediate Bax and Bak oligomerization. Recent reports have described a probable additional interface in Bak oligomers, formed by the C-terminus of α3 and α5 helices [22]. Furthermore, an α9:α9 interaction has been reported for Bax and Bak [23,59]. Finally, our present results in living cells would also accommodate a new model in which heterogeneous complexes would assemble through several interaction interfaces [25].

## 5. Conclusions

Present results using the BiFC technique indicate that interactions between members of the Bcl-2 family could be more promiscuous than usually accepted. Furthermore, our results suggest that Bax and Bak could be activated by BH3 proteins in a different way: BH3-only proteins bind to the H1α of Bax, while the canonic hydrophobic groove of Bak seems to be the site of binding for Bim and Puma. Concerning Bax and Bak oligomerization, our data would fit with models of symmetric BH3-in-groove dimers. Several interfaces could then be involved in the assembly of high-order oligomers. The findings of this study have to be seen in the light of some limitations. As with other techniques for this study of protein–protein interactions, BiFC has some drawbacks that have to be borne in mind. First, as this technique is based on the exogenous expression of protein fusions, it cannot be excluded that endogenous proteins do not behave in the same way. Another limitation concerns the possible effect of the fused fluorescent fragments on the function of the proteins. Finally, complementation could occur when the fusions are placed together in small subcellular compartments, even if there is no direct interaction. Although we have tried to minimize these limitations, and many of our results agree with previous findings using different methods, further studies would be necessary to fully understand the complex network of interactions among proteins of the Bcl-2 family in a cellular context.

## Data Availability

Not applicable.

## Supplementary Materials

The following supporting information can be downloaded at: https://www.mdpi.com/article/10.3390/cells12050800/s1, Figure S1: Two examples of the analysis of BiFC by flow cytometry; Figure S2: HeLa cells were cotransfected with the indicated BiFC fusions and expression was analyzed by Western Blot with anti-HA and anti-Flag tags; Figure S3: Multicolor data analysis example; Figure S4: Possible dimerization pairs through the BH3-in Groove and α1:α6 interfaces; Supplementary data: Nucleotide sequences of the fusions used in BiFC experiments.
