# Supplementary material for: Study of the Bcl-2 Interactome by BiFC Reveals Differences in the Activation Mechanism of Bax and Bak
Source: Cells. 2023 Mar 3;12(5):800. doi: 10.3390/cells12050800 (PMC10000386; doi:10.3390/cells12050800)
Supplement: Supplementary file 1 [file cells-12-00800-s001.zip › cells-2106357-supplementary.pdf]

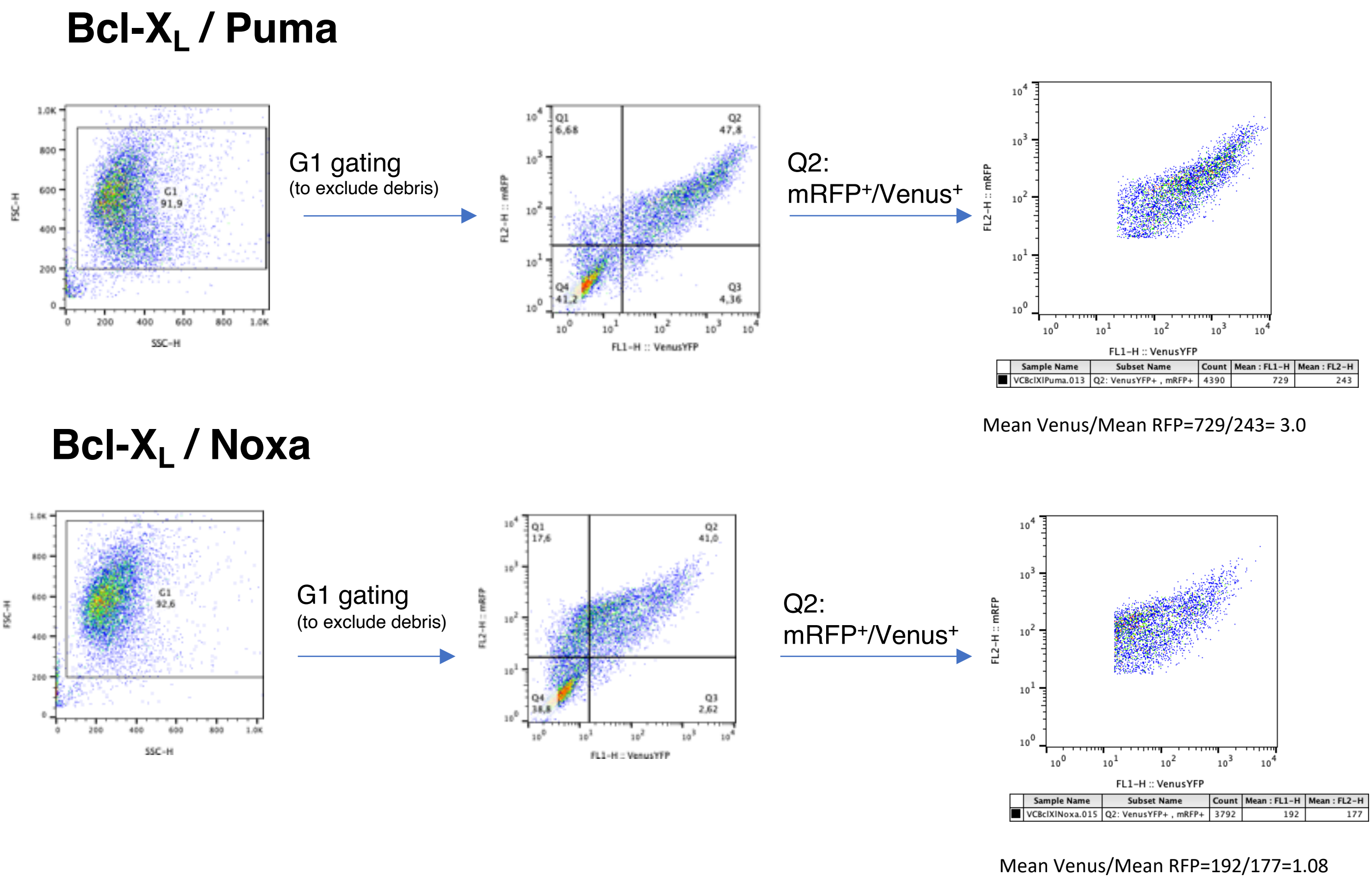

Figure S1- Two examples of the analysis of BiFC by flow cytometry. Mean Venus and RFP fluorescence intensities were determined in the double positive population and the Venus/mRFP ratio was calculated for each pair.

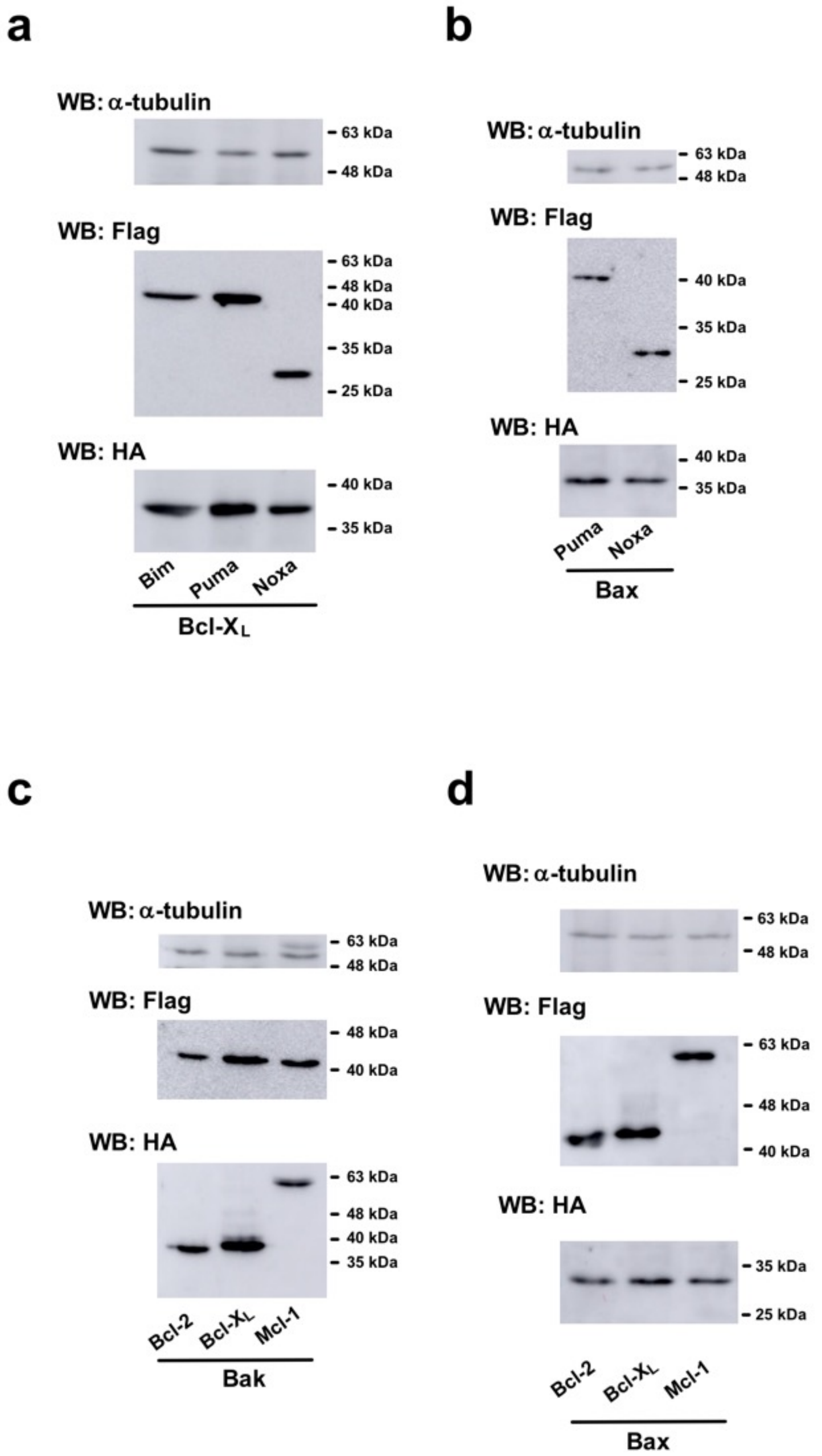

Figure S2- HeLa cells were cotransfected with the indicated BiFC fusions and expression was analyzed by Western Blot with anti-HA and anti-Flag tags.

a

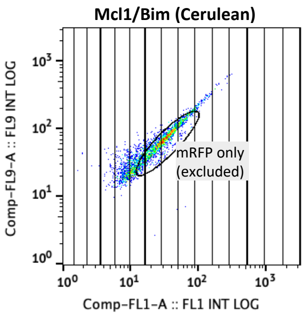

	Mcl-1/Bim/Bax		
	FL-1 Mean	FL-9 Mean	Count
R5	14,5	52,5	47
R6	19,9	52,5	152
R7	24,7	55,9	246
R8	33,6	71,9	70
R9	44,5	85,2	29
R10	57,5	107,8	16

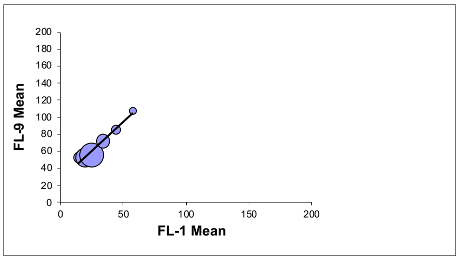

b

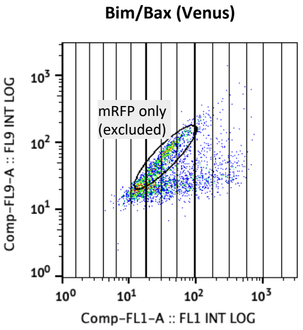

	Bim/Bax		
	FL-1 Mean	FL-9 Mean	Count
R6	34,8	32,9	327
R7	46,3	32,4	224
R8	62,7	30,3	159
R9	82,1	31,6	125
R10	110,1	31,5	87
R11	146,4	31,7	86
R12	196,2	34,4	58

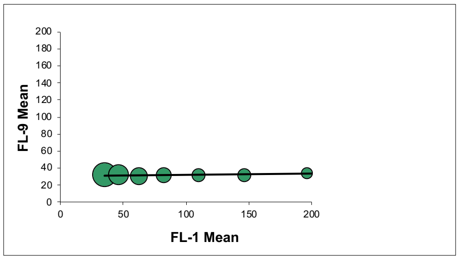

c

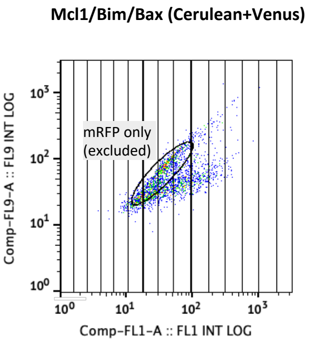

	Mcl-1/Bim/Bax		
	FL-1 Mean	FL-9 Mean	Count
R6	30,4	36,4	457
R7	45	44,6	368
R8	69,3	48,7	262
R9	110,4	50,2	143
R10	174,8	59,4	90

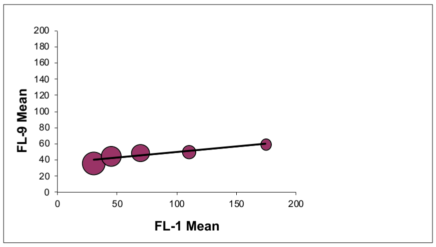

d

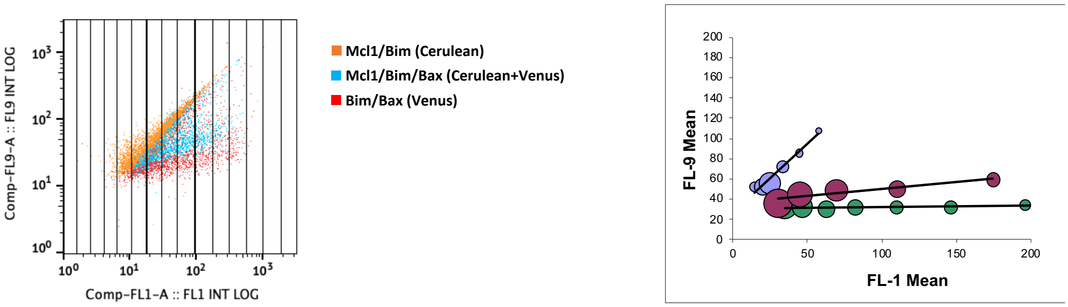

Figure S3- Multicolor data analysis example. HeLa cells were transfected with vectors expressing the CN-, VN- or CC-fusions for Mcl-1 and Bim (a), Bim and Bax (b) or Mcl-1, Bim and Bax(c), together with the pAL2-Myc-mRFP vector. After 24 hours, cerulean and Venus fluorescence signals were analyzed in the mRFP positive gated population by flow cytometry. FL-9 (cerulean)/FL-1 (Venus) histograms were divided in 15 sections in the FL-1 dimension, mean Venus and Cerulean fluorescence in each section were recorded (tables in the middle panel) and represented as a XY graphic and adjusted to a linear function using GraphPad Prism software (right panel). A control of cells transfected only with mRFP was performed and cells that were only positive for mRFP (ovals) were excluded for the analysis. Circle diameter is proportional to cell density (count) in the corresponding section of the histogram. (d) Overlay of the three histograms and the three graphics.

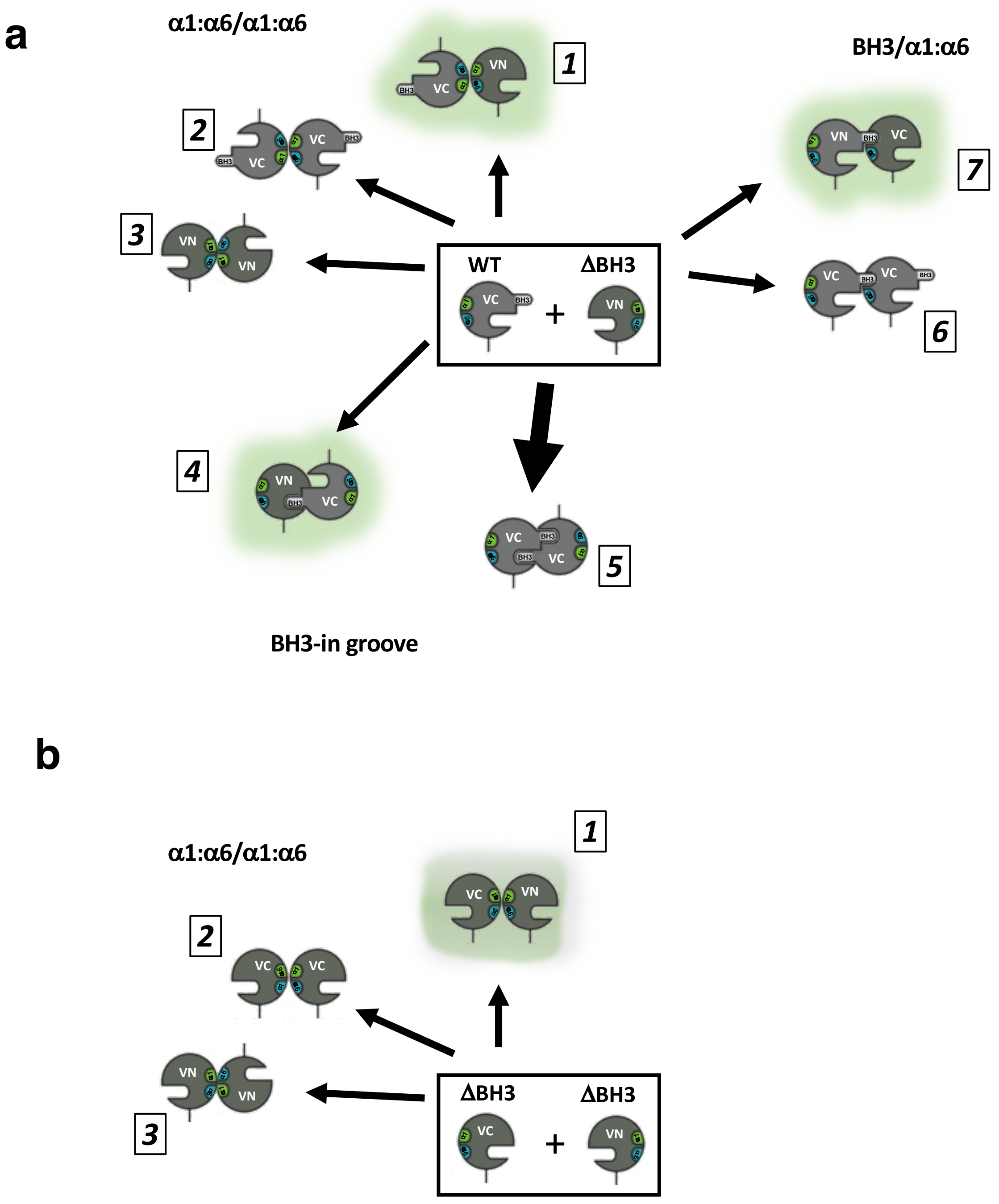

Figure S4- Possible dimerization pairs through the BH3-in Groove and α1:α6 interfaces. **(a)** Wild-type+ΔBH3 mutant. **(b)** ΔBH3 mutant+ΔBH3mutant. Pairs that allow for Venus complementation are highlighted in green. Dimerization of Bax chains fused to the same Venus half would reduce the yield of complementation.

Supplementary data (Gonzalo et al).

Nucleotide sequences of the fusions used in BiFC experiments

pBiFC-VN-Bcl2

ATGGACTACAAAGACGATGACGACAAGCTTGCGGCCGCATCGATAATGGTGAGCAAGGGCG
AGGAGCTGTTCACCGGGGTGGTGCCCATCCTGGTCGAGCTGGACGGCGACGTAAACGGCCA
CAAGTTCAGCGTGTCCGGCGAGGGCGAGGGCGATGCCACCTACGGCAAGCTGACCCTGAAG
CTGATCTGCACCACCGGCAAGCTGCCCGTGCCCTGGCCCACCCTCGTGACCACCCTGGGCTA
CGGCCTGCAGTGCTTCGCCCGCTACCCCGACCACATGAAGCAGCACGACTTCTTCAAGTCCG
CCATGCCCGAAGGCTACGTCCAGGAGCGCACCATCTTCTTCAAGGACGACGGCAACTACAA
GACCCGCGCCGAGGTGAAGTTCGAGGGCGACACCCTGGTGAACCGCATCGAGCTGAAGGGC
ATCGACTTCAAGGAGGACGGCAACATCCTGGGGCACAAGCTGGAGTACAACTACAACAGCC
ACAACGTCTATATCACCGCCGACAAGCAGAAGAACGGCATCAAGGCCAACTTCAAGATCCG
CCACAACATCGAGAGATCCATCGCCACCGCTAGCATGGCGCACGCTGGGAGAACAGGGTAC
GATAACCGGGAGATAGTGATGAAGTACATCCATTATAAGCTGTCGCAGAGGGGCTACGAGT
GGGATGCGGGAGATGTGGGCGCCGCGCCCCCGGGGGCCGCCCCCGCGCCGGGCATCTTCTC
CTCGCAGCCCGGGCACACGCCCCATACAGCCGCATCCCGGGACCCGGTCGCCAGGACCTCG
CCGCTGCAGACCCCGGCTGCCCCCGGCGCCGCCGCGGGGCCTGCGCTCAGCCCGGTGCCACC
TGTGGTCCACCTGACCCTCCGCCAGGCCGGCGACGACTTCTCCCGCCGCTACCGCCGCGACT
TCGCCGAGATGTCCAGGCAGCTGCACCTGACGCCCTTCACCGCGCGGGGACGCTTTGCCACG
GTGGTGGAGGAGCTCTTCAGGGACGGGGTGAACTGGGGGAGGATTGTGGCCTTCTTTGAGTT
CGGTGGGGTCATGTGTGTGGAGAGCGTCAACCGGGAGATGTCGCCCCTGGTGGACAACATC
GCCCTGTGGATGACTGAGTACCTGAACCGGCACCTGCACACCTGGATCCAGGATAACGGAG
GCTGGGATGCCTTTGTGGAACTGTACGGCCCCAGCATGCGGCCTCTGTTTGATTTCTCCTGGC
TGTCTCTGAAGACTCTGCTCAGTTTGGCCCTGGTGGGAGCTTGCATCACCCTGGGTGCCTATC
TGGGCCACAAGTGA

pBiFC-VC-Bcl2

ATGTACCCATACGATGTTCCAGATTACGCTCTTATGGCCATGGAGGCCCGAATTCGGGACAA
GCAGAAGAACGGCATCAAGGCCAACTTCAAGATCCGCCACAACATCGAGGACGGCGGCGTG
CAGCTCGCCGACCACTACCAGCAGAACACCCCCATCGGCGACGGCCCCGTGCTGCTGCCCGA
CAACCACTACCTGAGCTACCAGTCCGCCCTGAGCAAAGACCCCAACGAGAAGCGCGATCAC
ATGGTCCTGCTGGAGTTCGTGACCGCCGCCGGGATCACTCTCGGCATGGACGAGCTGTACAA
GGGTACCCGTCCGGCGTGCAAAATCCCGAACGACCTGAAACAGAAAGTCATGAACCACATC
GATATGGCGCACGCTGGGAGAACAGGGTACGATAACCGGGAGATAGTGATGAAGTACATCC
ATTATAAGCTGTCGCAGAGGGGCTACGAGTGGGATGCGGGAGATGTGGGCGCCGCGCCCCC
GGGGGCCGCCCCCGCGCCGGGCATCTTCTCCTCGCAGCCCGGGCACACGCCCCATACAGCCG
CATCCCGGGACCCGGTCGCCAGGACCTCGCCGCTGCAGACCCCGGCTGCCCCCGGCGCCGCC
GCGGGGCCTGCGCTCAGCCCGGTGCCACCTGTGGTCCACCTGACCCTCCGCCAGGCCGGCGA
CGACTTCTCCCGCCGCTACCGCCGCGACTTCGCCGAGATGTCCAGGCAGCTGCACCTGACGC
CCTTCACCGCGCGGGGACGCTTTGCCACGGTGGTGGAGGAGCTCTTCAGGGACGGGGTGAA
CTGGGGGAGGATTGTGGCCTTCTTTGAGTTCGGTGGGGTCATGTGTGTGGAGAGCGTCAACC
GGGAGATGTCGCCCCTGGTGGACAACATCGCCCTGTGGATGACTGAGTACCTGAACCGGCA
CCTGCACACCTGGATCCAGGATAACGGAGGCTGGGATGCCTTTGTGGAACTGTACGGCCCCA
GCATGCGGCCTCTGTTTGATTTCTCCTGGCTGTCTCTGAAGACTCTGCTCAGTTTGGCCCTGG
TGGGAGCTTGCATCACCCTGGGTGCCTATCTGGGCCACAAGTGA

pBiFC-VN-BclX$_L$

ATGGACTACAAAGACGATGACGACAAGCTTGCGGCCGCGAATTCAATGGTGAGCAAGGGCG
AGGAGCTGTTCACCGGGGTGGTGCCCATCCTGGTCGAGCTGGACGGCGACGTAAACGGCCA
CAAGTTCAGCGTGTCCGGCGAGGGCGAGGGCGATGCCACCTACGGCAAGCTGACCCTGAAG
CTGATCTGCACCACCGGCAAGCTGCCCGTGCCCTGGCCCACCCTCGTGACCACCCTGGGCTA
CGGCCTGCAGTGCTTCGCCCGCTACCCCGACCACATGAAGCAGCACGACTTCTTCAAGTCCG
CCATGCCCGAAGGCTACGTCCAGGAGCGCACCATCTTCTTCAAGGACGACGGCAACTACAA
GACCCGCGCCGAGGTGAAGTTCGAGGGCGACACCCTGGTGAACCGCATCGAGCTGAAGGGC
ATCGACTTCAAGGAGGACGGCAACATCCTGGGGCACAAGCTGGAGTACAACTACAACAGCC
ACAACGTCTATATCACCGCCGACAAGCAGAAGAACGGCATCAAGGCCAACTTCAAGATCCG
CCACAACATCGAGAGATCCATCGCCACCGCTAGCATGTCTCAGAGCAACCGGGAGCTGGTG
GTTGACTTTCTCTCCTACAAGCTTTCCCAGAAAGGATACAGCTGGAGTCAGTTTAGTGATGT
GGAAGAGAACAGGACTGAGGCCCCAGAAGGGACTGAATCGGAGATGGAGACCCCCAGTGC
CATCAATGGCAACCCATCCTGGCACCTGGCAGACAGCCCCGCGGTGAATGGAGCCACTGGC
CACAGCAGCAGTTTGGATGCCCGGGAGGTGATCCCCATGGCAGCAGTAAAGCAAGCGCTGA
GGGAGGCAGGCGACGAGTTTGAACTGCGGTACCGGCGGGCATTCAGTGACCTGACATCCCA
GCTCCACATCACCCCAGGGACAGCATATCAGAGCTTTGAACAGGTAGTGAATGAACTCTTCC
GGGATGGGGTAAACTGGGGTCGCATTGTGGCCTTTTTCTCCTTCGGCGGGGCACTGTGCGTG
GAAAGCGTAGACAAGGAGATGCAGGTATTGGTGAGTCGGATCGCAGCTTGGATGGCCACTT
ACCTGAATGACCACCTAGAGCCTTGGATCCAGGAGAACGGCGGCTGGGATACTTTTGTGGA
ACTCTATGGGAACAATGCAGCAGCCGAGAGCCGAAAGGGCCAGGAACGCTTCAACCGCTGG
TTCCTGACGGGCATGACTGTGGCCGGCGTGGTTCTGCTGGGCTCACTCTTCAGTCGGAAATG
A

pBiFC-VC-BclX$_L$

ATGTACCCATACGATGTTCCAGATTACGCTCTTATGGCCATGGAGGCCCGAATTCGGGACAA
GCAGAAGAACGGCATCAAGGCCAACTTCAAGATCCGCCACAACATCGAGGACGGCGGCGTG
CAGCTCGCCGACCACTACCAGCAGAACACCCCCATCGGCGACGGCCCCGTGCTGCTGCCCGA
CAACCACTACCTGAGCTACCAGTCCGCCCTGAGCAAAGACCCCAACGAGAAGCGCGATCAC
ATGGTCCTGCTGGAGTTCGTGACCGCCGCCGGGATCACTCTCGGCATGGACGAGCTGTACAA
GGGTACCCGTCCGGCGTGCAAAATCCCGAACGACCTGAAACAGAAAGTCATGAACCACATC
GATATGTCTCAGAGCAACCGGGAGCTGGTGGTTGACTTTCTCTCCTACAAGCTTTCCCAGAA
AGGATACAGCTGGAGTCAGTTTAGTGATGTGGAAGAGAACAGGACTGAGGCCCCAGAAGGG
ACTGAATCGGAGATGGAGACCCCCAGTGCCATCAATGGCAACCCATCCTGGCACCTGGCAG
ACAGCCCCGCGGTGAATGGAGCCACTGGCCACAGCAGCAGTTTGGATGCCCGGGAGGTGAT
CCCCATGGCAGCAGTAAAGCAAGCGCTGAGGGAGGCAGGCGACGAGTTTGAACTGCGGTAC
CGGCGGGCATTCAGTGACCTGACATCCCAGCTCCACATCACCCCAGGGACAGCATATCAGA
GCTTTGAACAGGTAGTGAATGAACTCTTCCGGGATGGGGTAAACTGGGGTCGCATTGTGGCC
TTTTTCTCCTTCGGCGGGGCACTGTGCGTGGAAAGCGTAGACAAGGAGATGCAGGTATTGGT
GAGTCGGATCGCAGCTTGGATGGCCACTTACCTGAATGACCACCTAGAGCCTTGGATCCAGG
AGAACGGCGGCTGGGATACTTTTGTGGAACTCTATGGGAACAATGCAGCAGCCGAGAGCCG
AAAGGGCCAGGAACGCTTCAACCGCTGGTTCCTGACGGGCATGACTGTGGCCGGCGTGGTTC
TGCTGGGCTCACTCTTCAGTCGGAAATGA

pBiFC-VN-Mcl1

ATGGACTACAAAGACGATGACGACAAGCTTGCGGCCGCGAATTCAATGGTGAGCAAGGGCG
AGGAGCTGTTCACCGGGGTGGTGCCCATCCTGGTCGAGCTGGACGGCGACGTAAACGGCCA
CAAGTTCAGCGTGTCCGGCGAGGGCGAGGGCGATGCCACCTACGGCAAGCTGACCCTGAAG
CTGATCTGCACCACCGGCAAGCTGCCCGTGCCCTGGCCCACCCTCGTGACCACCCTGGGCTA
CGGCCTGCAGTGCTTCGCCCGCTACCCCGACCACATGAAGCAGCACGACTTCTTCAAGTCCG
CCATGCCCGAAGGCTACGTCCAGGAGCGCACCATCTTCTTCAAGGACGACGGCAACTACAA
GACCCGCGCCGAGGTGAAGTTCGAGGGCGACACCCTGGTGAACCGCATCGAGCTGAAGGGC
ATCGACTTCAAGGAGGACGGCAACATCCTGGGGCACAAGCTGGAGTACAACTACAACAGCC
ACAACGTCTATATCACCGCCGACAAGCAGAAGAACGGCATCAAGGCCAACTTCAAGATCCG
CCACAACATCGAGAGATCCATCGCCACCGCTAGCATGTTTGGCCTCAAAAGAAACGCGGTA
ATCGGACTCAACCTCTACTGTGGGGGGGCCGGCTTGGGGGCCGGCAGCGGCGGCGCCACCC
GCCCGGGAGGGCGACTTTTGGCTACGGAGAAGGAGGCCTCGGCCCGGCGAGAGATAGGGGG
AGGGGAGGCCGGCGCGGTGATTGGCGGAAGCGCCGGCGCAAGCCCCCCGTCCACCCTCACG
CCAGACTCCCGGAGGGTCGCGCGGCCGCCGCCCATTGGCGCCGAGGTCCCCGACGTCACCG
CGACCCCCGCGAGGCTGCTTTTCTTCGCGCCCACCCGCCGCGCGGCGCCGCTTGAGGAGATG
GAAGCCCCGGCCGCTGACGCCATCATGTCGCCCGAAGAGGAGCTGGACGGGTACGAGCCGG
AGCCTCTCGGGAAGCGGCCGGCTGTCCTGCCGCTGCTGGAGTTGGTCGGGGAATCTGGTAAT
AACACCAGTACGGACGGGTCACTACCCTCGACGCCGCCGCCAGCAGAGGAGGAGGAGGACG
AGTTGTACCGGCAGTCGCTGGAGATTATCTCTCGGTACCTTCGGGAGCAGGCCACCGGCGCC
AAGGACACAAAGCCAATGGGCAGGTCTGGGGCCACCAGCAGGAAGGCGCTGGAGACCTTAC
GACGGGTTGGGGATGGCGTGCAGCGCAACCACGAGACGGCCTTCCAAGGCATGCTTCGGAA
ACTGGACATCAAAAACGAAGACGATGTGAAATCGTTGTCTCGAGTGATGATCCATGTTTTCA
GCGACGGCGTAACAAACTGGGGCAGGATTGTGACTCTCATTTCTTTTGGTGCCTTTGTGGCT
AAACACTTGAAGACCATAAACCAAGAAAGCTGCATCGAACCATTAGCAGAAAGTATCACAG
ACGTTCTCGTAAGGACAAAACGGGACTGGCTAGTTAAACAAAGAGGCTGGGATGGGTTTGT
GGAGTTCTTCCATGTAGAGGACCTAGAAGGTGGCATCAGGAATGTGCTGCTGGCTTTTGCAG
GTGTTGCTGGAGTAGGAGCTGGTTTGGCATATCTAATAAGATAG

pBiFC-VC-Mcl1

ATGTACCCATACGATGTTCCAGATTACGCTCTTATGGCCATGGAGGCCCGAATTCGGGACAA
GCAGAAGAACGGCATCAAGGCCAACTTCAAGATCCGCCACAACATCGAGGACGGCGGCGTG
CAGCTCGCCGACCACTACCAGCAGAACACCCCCATCGGCGACGGCCCCGTGCTGCTGCCCGA
CAACCACTACCTGAGCTACCAGTCCGCCCTGAGCAAAGACCCCAACGAGAAGCGCGATCAC
ATGGTCCTGCTGGAGTTCGTGACCGCCGCCGGGATCACTCTCGGCATGGACGAGCTGTACAA
GGGTACCCGTCCGGCGTGCAAAATCCCGAACGACCTGAAACAGAAAGTCATGAACCACATC
GATATGTTTGGCCTCAAAAGAAACGCGGTAATCGGACTCAACCTCTACTGTGGGGGGGCCG
GCTTGGGGGCCGGCAGCGGCGGCGCCACCCGCCCGGGAGGGCGACTTTTGGCTACGGAGAA
GGAGGCCTCGGCCCGGCGAGAGATAGGGGGAGGGGAGGCCGGCGCGGTGATTGGCGGAAG
CGCCGGCGCAAGCCCCCCGTCCACCCTCACGCCAGACTCCCGGAGGGTCGCGCGGCCGCCG
CCCATTGGCGCCGAGGTCCCCGACGTCACCGCGACCCCCGCGAGGCTGCTTTTCTTCGCGCC
CACCCGCCGCGCGGCGCCGCTTGAGGAGATGGAAGCCCCGGCCGCTGACGCCATCATGTCG
CCCGAAGAGGAGCTGGACGGGTACGAGCCGGAGCCTCTCGGGAAGCGGCCGGCTGTCCTGC
CGCTGCTGGAGTTGGTCGGGGAATCTGGTAATAACACCAGTACGGACGGGTCACTACCCTCG
ACGCCGCCGCCAGCAGAGGAGGAGGAGGACGAGTTGTACCGGCAGTCGCTGGAGATTATCT
CTCGGTACCTTCGGGAGCAGGCCACCGGCGCCAAGGACACAAAGCCAATGGGCAGGTCTGG
GGCCACCAGCAGGAAGGCGCTGGAGACCTTACGACGGGTTGGGGATGGCGTGCAGCGCAAC
CACGAGACGGCCTTCCAAGGCATGCTTCGGAAACTGGACATCAAAAACGAAGACGATGTGA
AATCGTTGTCTCGAGTGATGATCCATGTTTTCAGCGACGGCGTAACAAACTGGGGCAGGATT
GTGACTCTCATTTCTTTTGGTGCCTTTGTGGCTAAACACTTGAAGACCATAAACCAAGAAAG
CTGCATCGAACCATTAGCAGAAAGTATCACAGACGTTCTCGTAAGGACAAAACGGGACTGG
CTAGTTAAACAAAGAGGCTGGGATGGGTTTGTGGAGTTCTTCCATGTAGAGGACCTAGAAG
GTGGCATCAGGAATGTGCTGCTGGCTTTTGCAGGTGTTGCTGGAGTAGGAGCTGGTTTGGCA
TATCTAATAAGATAG

pBiFC-VN-Bim

ATGGACTACAAAGACGATGACGACAAGCTTGCGGCCGCGAATTCAATGGTGAGCAAGGGCG
AGGAGCTGTTCACCGGGGTGGTGCCCATCCTGGTCGAGCTGGACGGCGACGTAAACGGCCA
CAAGTTCAGCGTGTCCGGCGAGGGCGAGGGCGATGCCACCTACGGCAAGCTGACCCTGAAG
CTGATCTGCACCACCGGCAAGCTGCCCGTGCCCTGGCCCACCCTCGTGACCACCCTGGGCTA
CGGCCTGCAGTGCTTCGCCCGCTACCCCGACCACATGAAGCAGCACGACTTCTTCAAGTCCG
CCATGCCCGAAGGCTACGTCCAGGAGCGCACCATCTTCTTCAAGGACGACGGCAACTACAA
GACCCGCGCCGAGGTGAAGTTCGAGGGCGACACCCTGGTGAACCGCATCGAGCTGAAGGGC
ATCGACTTCAAGGAGGACGGCAACATCCTGGGGCACAAGCTGGAGTACAACTACAACAGCC
ACAACGTCTATATCACCGCCGACAAGCAGAAGAACGGCATCAAGGCCAACTTCAAGATCCG
CCACAACATCGAGAGATCCATCGCCACCGCTAGCATGGCAAAGCAACCTTCTGATGTAAGTT
CTGAGTGTGACCGAGAAGGTAGACAATTGCAGCCTGCGGAGAGGCCTCCCCAGCTCAGACC
TGGGGCCCCTACCTCCCTACAGACAGAGCCACAAGGTAATCCTGAAGGCAATCACGGAGGT
GAAGGGGACAGCTGCCCCCACGGCAGCCCTCAGGGCCCGCTGGCCCCACCTGCCAGCCCTG
GCCCTTTTGCTACCAGATCCCCGCTTTTCATCTTTATGAGAAGATCCTCCCTGCTGTCTCGAT
CCTCCAGTGGGTATTTCTCTTTTGACACAGACAGGAGCCCAGCACCCATGAGTTGTGACAAA
TCAACACAAACCCCAAGTCCTCCTTGCCAGGCCTTCAACCACTATCTCAGTGCAATGGCTTC
CATGAGGCAGGCTGAACCTGCAGATATGCGCCCAGAGATATGGATCGCCCAAGAGTTGCGG
CGTATCGGAGACGAGTTTAACGCTTACTATGCAAGGAGGGTATTTTTGAATAATTACCAAGC
AGCCGAAGACCACCCACGAATGGTTATCTTACGACTGTTACGTTACATTGTCCGCCTGGTGT
GGAGAATGCATTGA

pBiFC-VC-Bim

ATGTACCCATACGATGTTCCAGATTACGCTCTTATGGCCATGGAGGCCCGAATTCGGGACAA
GCAGAAGAACGGCATCAAGGCCAACTTCAAGATCCGCCACAACATCGAGGACGGCGGCGTG
CAGCTCGCCGACCACTACCAGCAGAACACCCCCATCGGCGACGGCCCCGTGCTGCTGCCCGA
CAACCACTACCTGAGCTACCAGTCCAAACTGAGCAAAGACCCCAACGAGAAGCGCGATCAC
ATGGTCCTGCTGGAGTTCGTGACCGCCGCCGGGATCACTCTCGGCATGGACGAGCTGTACAA
GGGTACCCGTCCGGCGTGCAAAATCCCGAACGACCTGAAACAGAAAGTCATGAACCACATC
GATATGGCAAAGCAACCTTCTGATGTAAGTTCTGAGTGTGACCGAGAAGGTAGACAATTGC
AGCCTGCGGAGAGGCCTCCCCAGCTCAGACCTGGGGCCCCTACCTCCCTACAGACAGAGCC
ACAAGGTAATCCTGAAGGCAATCACGGAGGTGAAGGGGACAGCTGCCCCCACGGCAGCCCT
CAGGGCCCGCTGGCCCCACCTGCCAGCCCTGGCCCTTTTGCTACCAGATCCCCGCTTTTCATC
TTTATGAGAAGATCCTCCCTGCTGTCTCGATCCTCCAGTGGGTATTTCTCTTTTGACACAGAC
AGGAGCCCAGCACCCATGAGTTGTGACAAATCAACACAAACCCCAAGTCCTCCTTGCCAGG
CCTTCAACCACTATCTCAGTGCAATGGCTTCCATGAGGCAGGCTGAACCTGCAGATATGCGC
CCAGAGATATGGATCGCCCAAGAGTTGCGGCGTATCGGAGACGAGTTTAACGCTTACTATGC
AAGGAGGGTATTTTTGAATAATTACCAAGCAGCCGAAGACCACCCACGAATGGTTATCTTAC
GACTGTTACGTTACATTGTCCGCCTGGTGTGGAGAATGCATTGA

pBiFC-VN-PUMA

ATGGACTACAAAGACGATGACGACAAGCTTGCGGCCGCGAATTCAATGGTGAGCAAGGGCG
AGGAGCTGTTCACCGGGGTGGTGCCCATCCTGGTCGAGCTGGACGGCGACGTAAACGGCCA
CAAGTTCAGCGTGTCCGGCGAGGGCGAGGGCGATGCCACCTACGGCAAGCTGACCCTGAAG
CTGATCTGCACCACCGGCAAGCTGCCCGTGCCCTGGCCCACCCTCGTGACCACCCTGGGCTA
CGGCCTGCAGTGCTTCGCCCGCTACCCCGACCACATGAAGCAGCACGACTTCTTCAAGTCCG
CCATGCCCGAAGGCTACGTCCAGGAGCGCACCATCTTCTTCAAGGACGACGGCAACTACAA
GACCCGCGCCGAGGTGAAGTTCGAGGGCGACACCCTGGTGAACCGCATCGAGCTGAAGGGC
ATCGACTTCAAGGAGGACGGCAACATCCTGGGGCACAAGCTGGAGTACAACTACAACAGCC
ACAACGTCTATATCACCGCCGACAAGCAGAAGAACGGCATCAAGGCCAACTTCAAGATCCG
CCACAACATCGAGAGATCCATCGCCACCGCTAGCATGGCCCGCGCACGCCAGGAGGGCAGC
TCCCCGGAGCCCGTAGAGGGCCTGGCCCGCGACGGCCCGCGCCCCTTCCCGCTCGGCCGCCT
GGTGCCCTCGGCAGTGTCCTGCGGCCTCTGCGAGCCCGGCCTGGCTGCCGCCCCCGCCGCCC
CCACCCTGCTGCCCGCTGCCTACCTCTGCGCCCCCACCGCCCCACCCGCCGTCACCGCCGCCC
TGGGGGGTTCCCGCTGGCCTGGGGGTCCCCGCAGCCGGCCCCGAGGCCCGCGCCCGGACGG
TCCTCAGCCCTCGCTCTCGCTGGCGGAGCAGCACCTGGAGTCGCCCGTGCCCAGCGCCCCGG
GGGCTCTGGCGGGCGGTCCCACCCAGGCGGCCCCGGGAGTCCGCGGGGAGGAGGAACAGTG
GGCCCGGGAGATCGGGGCCCAGCTGCGGCGGATGGCGGACGACCTCAACGCACAGTACGAG
CGGCGGAGACAAGAGGAGCAGCAGCGGCACCGCCCCTCACCCTGGAGGGTCCTGTACAATC
TCATCATGGGACTCCTGCCCTTACCCAGGGGCCACAGAGCCCCCGAGATGGAGCCCAATTGA

pBiFC-VC-PUMA

ATGTACCCATACGATGTTCCAGATTACGCTCTTATGGCCATGGAGGCCCGAATTCGGGACAA
GCAGAAGAACGGCATCAAGGCCAACTTCAAGATCCGCCACAACATCGAGGACGGCGGCGTG
CAGCTCGCCGACCACTACCAGCAGAACACCCCCATCGGCGACGGCCCCGTGCTGCTGCCCGA
CAACCACTACCTGAGCTACCAGTCCAAACTGAGCAAAGACCCCAACGAGAAGCGCGATCAC
ATGGTCCTGCTGGAGTTCGTGACCGCCGCCGGGATCACTCTCGGCATGGACGAGCTGTACAA
GGGTACCCGTCCGGCGTGCAAAATCCCGAACGACCTGAAACAGAAAGTCATGAACCACATC
GATATGGCCCGCGCACGCCAGGAGGGCAGCTCCCCGGAGCCCGTAGAGGGCCTGGCCCGCG
ACGGCCCGCGCCCCTTCCCGCTCGGCCGCCTGGTGCCCTCGGCAGTGTCCTGCGGCCTCTGC
GAGCCCGGCCTGGCTGCCGCCCCCGCCGCCCCCACCCTGCTGCCCGCTGCCTACCTCTGCGC
CCCCACCGCCCCACCCGCCGTCACCGCCGCCCTGGGGGGTTCCCGCTGGCCTGGGGGTCCCC
GCAGCCGGCCCCGAGGCCCGCGCCCGGACGGTCCTCAGCCCTCGCTCTCGCTGGCGGAGCA
GCACCTGGAGTCGCCCGTGCCCAGCGCCCCGGGGGCTCTGGCGGGCGGTCCCACCCAGGCG
GCCCCGGGAGTCCGCGGGGAGGAGGAACAGTGGGCCCGGGAGATCGGGGCCCAGCTGCGG
CGGATGGCGGACGACCTCAACGCACAGTACGAGCGGCGGAGACAAGAGGAGCAGCAGCGG
CACCGCCCCTCACCCTGGAGGGTCCTGTACAATCTCATCATGGGACTCCTGCCCTTACCCAG
GGGCCACAGAGCCCCCGAGATGGAGCCCAATTGA

pBiFC-VN-Noxa

ATGGACTACAAAGACGATGACGACAAGCTTGCGGCCGCGAATTCAATGGTGAGCAAGGGCG
AGGAGCTGTTCACCGGGGTGGTGCCCATCCTGGTCGAGCTGGACGGCGACGTAAACGGCCA
CAAGTTCAGCGTGTCCGGCGAGGGCGAGGGCGATGCCACCTACGGCAAGCTGACCCTGAAG
CTGATCTGCACCACCGGCAAGCTGCCCGTGCCCTGGCCCACCCTCGTGACCACCCTGGGCTA
CGGCCTGCAGTGCTTCGCCCGCTACCCCGACCACATGAAGCAGCACGACTTCTTCAAGTCCG
CCATGCCCGAAGGCTACGTCCAGGAGCGCACCATCTTCTTCAAGGACGACGGCAACTACAA
GACCCGCGCCGAGGTGAAGTTCGAGGGCGACACCCTGGTGAACCGCATCGAGCTGAAGGGC
ATCGACTTCAAGGAGGACGGCAACATCCTGGGGCACAAGCTGGAGTACAACTACAACAGCC
ACAACGTCTATATCACCGCCGACAAGCAGAAGAACGGCATCAAGGCCAACTTCAAGATCCG
CCACAACATCGAGAGATCCATCGCCACCGCTAGCATGCCTGGGAAGAAGGCGCGCAAGAAC
GCTCAACCGAGCCCCGCGCGGGCTCCAGCAGAGCTGGAAGTCGAGTGTGCTACTCAACTCA
GGAGATTTGGAGACAAACTGAACTTCCGGCAGAAACTTCTGAATCTGATATCCAAACTCTTC
TGCTCAGGAACCTGA

pBiFC-VC-Noxa

ECO RI / CLA I/ BGL II

ATGTACCCATACGATGTTCCAGATTACGCTCTTATGGCCATGGAGGCCCGAATTCGGGACAA
GCAGAAGAACGGCATCAAGGCCAACTTCAAGATCCGCCACAACATCGAGGACGGCGGCGTG
CAGCTCGCCGACCACTACCAGCAGAACACCCCCATCGGCGACGGCCCCGTGCTGCTGCCCGA
CAACCACTACCTGAGCTACCAGTCCAAACTGAGCAAAGACCCCAACGAGAAGCGCGATCAC
ATGGTCCTGCTGGAGTTCGTGACCGCCGCCGGGATCACTCTCGGCATGGACGAGCTGTACAA
GGGTACCCGTCCGGCGTGCAAAATCCCGAACGACCTGAAACAGAAAGTCATGAACCACATC
GATATGCCTGGGAAGAAGGCGCGCAAGAACGCTCAACCGAGCCCCGCGCGGGCTCCAGCAG
AGCTGGAAGTCGAGTGTGCTACTCAACTCAGGAGATTTGGAGACAAACTGAACTTCCGGCA
GAAACTTCTGAATCTGATATCCAAACTCTTCTGCTCAGGAACCTGA

pBiFC-VN-Bax

ATGGACTACAAAGACGATGACGACAAGCTTGCGGCCGCGAATTCAATGGTGAGCAAGGGCG
AGGAGCTGTTCACCGGGGTGGTGCCCATCCTGGTCGAGCTGGACGGCGACGTAAACGGCCA
CAAGTTCAGCGTGTCCGGCGAGGGCGAGGGCGATGCCACCTACGGCAAGCTGACCCTGAAG
CTGATCTGCACCACCGGCAAGCTGCCCGTGCCCTGGCCCACCCTCGTGACCACCCTGGGCTA
CGGCCTGCAGTGCTTCGCCCGCTACCCCGACCACATGAAGCAGCACGACTTCTTCAAGTCCG
CCATGCCCGAAGGCTACGTCCAGGAGCGCACCATCTTCTTCAAGGACGACGGCAACTACAA
GACCCGCGCCGAGGTGAAGTTCGAGGGCGACACCCTGGTGAACCGCATCGAGCTGAAGGGC
ATCGACTTCAAGGAGGACGGCAACATCCTGGGGCACAAGCTGGAGTACAACTACAACAGCC
ACAACGTCTATATCACCGCCGACAAGCAGAAGAACGGCATCAAGGCCAACTTCAAGATCCG
CCACAACATCGAGAGATCCATCGCCACCGCTAGCATGGACGGGTCCGGGGAGCAGCCCAGA
GGCGGGGGGCCCACCAGCTCTGAGCAGATCATGAAGACAGGGGCCCTTTTGCTTCAGGGTTT
CATCCAGGATCGAGCAGGGCGAATGGGGGGGGAGGCACCCGAGCTGGCCCTGGACCCGGTG
CCTCAGGATGCGTCCACCAAGAAGCTGAGCGAGTGTCTCAAGCGCATCGGGGACGAACTGG
ACAGTAACATGGAGCTGCAGAGGATGATTGCCGCCGTGGACACAGACTCCCCCCGAGAGGC
CTTTTTCCGAGTGGCAGCTGACATGTTTTCTGACGGCAACTTCAACTGGGGCCGGGTTGTCGC
CCTTTTCTACTTTGCCAGCAAACTGGTGCTCAAGGCCCTGTGCACCAAGGTGCCGGAACTGA
TCAGAACCATCATGGGCTGGACATTGGACTTCCTCCGGGAGCGGCTGTTGGGCTGGATCCAA
GACCAGGGTGGTTGGGACGGCCTCCTCTCCTACTTTGGGACGCCCACGTGGCAGACCGTGAC
CATCTTTGTGGCGGGAGTGCTCACCGCCTCACTCACCATCTGGAAGAAGATGGGCTGA

pBiFC-VC-Bax

ATGTACCCATACGATGTTCCAGATTACGCTCTTATGGCCATGGAGGCCCGAATTCGGGACAA
GCAGAAGAACGGCATCAAGGCCAACTTCAAGATCCGCCACAACATCGAGGACGGCGGCGTG
CAGCTCGCCGACCACTACCAGCAGAACACCCCCATCGGCGACGGCCCCGTGCTGCTGCCCGA
CAACCACTACCTGAGCTACCAGTCCAAACTGAGCAAAGACCCCAACGAGAAGCGCGATCAC
ATGGTCCTGCTGGAGTTCGTGACCGCCGCCGGGATCACTCTCGGCATGGACGAGCTGTACAA
GGGTACCCGTCCGGCGTGCAAAATCCCGAACGACCTGAAACAGAAAGTCATGAACCACATC
GATATGGACGGGTCCGGGGAGCAGCCCAGAGGCGGGGGGCCCACCAGCTCTGAGCAGATCA
TGAAGACAGGGGCCCTTTTGCTTCAGGGTTTCATCCAGGATCGAGCAGGGCGAATGGGGGG
GGAGGCACCCGAGCTGGCCCTGGACCCGGTGCCTCAGGATGCGTCCACCAAGAAGCTGAGC
GAGTGTCTCAAGCGCATCGGGGACGAACTGGACAGTAACATGGAGCTGCAGAGGATGATTG
CCGCCGTGGACACAGACTCCCCCCGAGAGGCCTTTTTCCGAGTGGCAGCTGACATGTTTTCT
GACGGCAACTTCAACTGGGGCCGGGTTGTCGCCCTTTTCTACTTTGCCAGCAAACTGGTGCT
CAAGGCCCTGTGCACCAAGGTGCCGGAACTGATCAGAACCATCATGGGCTGGACATTGGAC
TTCCTCCGGGAGCGGCTGTTGGGCTGGATCCAAGACCAGGGTGGTTGGGACGGCCTCCTCTC
CTACTTTGGGACGCCCACGTGGCAGACCGTGACCATCTTTGTGGCGGGAGTGCTCACCGCCT
CACTCACCATCTGGAAGAAGATGGGCTGA

pBiFC-VN-Bax Lys21Glu

ATGGACTACAAAGACGATGACGACAAGCTTGCGGCCGCGAATTCAATGGTGAGCAAGGGCG
AGGAGCTGTTCACCGGGGTGGTGCCCATCCTGGTCGAGCTGGACGGCGACGTAAACGGCCA
CAAGTTCAGCGTGTCCGGCGAGGGCGAGGGCGATGCCACCTACGGCAAGCTGACCCTGAAG
CTGATCTGCACCACCGGCAAGCTGCCCGTGCCCTGGCCCACCCTCGTGACCACCCTGGGCTA
CGGCCTGCAGTGCTTCGCCCGCTACCCCGACCACATGAAGCAGCACGACTTCTTCAAGTCCG
CCATGCCCGAAGGCTACGTCCAGGAGCGCACCATCTTCTTCAAGGACGACGGCAACTACAA
GACCCGCGCCGAGGTGAAGTTCGAGGGCGACACCCTGGTGAACCGCATCGAGCTGAAGGGC
ATCGACTTCAAGGAGGACGGCAACATCCTGGGGCACAAGCTGGAGTACAACTACAACAGCC
ACAACGTCTATATCACCGCCGACAAGCAGAAGAACGGCATCAAGGCCAACTTCAAGATCCG
CCACAACATCGAGAGATCCATCGCCACCGCTAGCATGGACGGGTCCGGGGAGCAGCCCAGA
GGCGGGGGGCCCACCAGCTCTGAGCAGATCATGGAGACAGGGGCCCTTTTGCTTCAGGGTTT
CATCCAGGATCGAGCAGGGCGAATGGGGGGGGAGGCACCCGAGCTGGCCCTGGACCCGGTG
CCTCAGGATGCGTCCACCAAGAAGCTGAGCGAGTGTCTCAAGCGCATCGGGGACGAACTGG
ACAGTAACATGGAGCTGCAGAGGATGATTGCCGCCGTGGACACAGACTCCCCCCGAGAGGC
CTTTTTCCGAGTGGCAGCTGACATGTTTTCTGACGGCAACTTCAACTGGGGCCGGGTTGTCGC
CCTTTTCTACTTTGCCAGCAAACTGGTGCTCAAGGCCCTGTGCACCAAGGTGCCGGAACTGA
TCAGAACCATCATGGGCTGGACATTGGACTTCCTCCGGGAGCGGCTGTTGGGCTGGATCCAA
GACCAGGGTGGTTGGGACGGCCTCCTCTCCTACTTTGGGACGCCCACGTGGCAGACCGTGAC
CATCTTTGTGGCGGGAGTGCTCACCGCCTCACTCACCATCTGGAAGAAGATGGGCTGA

pBiFC-VC-Bax Lys21Glu

ATGTACCCATACGATGTTCCAGATTACGCTCTTATGGCCATGGAGGCCCGAATTCGGGACAA
GCAGAAGAACGGCATCAAGGCCAACTTCAAGATCCGCCACAACATCGAGGACGGCGGCGTG
CAGCTCGCCGACCACTACCAGCAGAACACCCCCATCGGCGACGGCCCCGTGCTGCTGCCCGA
CAACCACTACCTGAGCTACCAGTCCAAACTGAGCAAAGACCCCAACGAGAAGCGCGATCAC
ATGGTCCTGCTGGAGTTCGTGACCGCCGCCGGGATCACTCTCGGCATGGACGAGCTGTACAA
GGGTACCCGTCCGGCGTGCAAAATCCCGAACGACCTGAAACAGAAAGTCATGAACCACATC
GATATGGACGGGTCCGGGGAGCAGCCCAGAGGCGGGGGGCCCACCAGCTCTGAGCAGATCA
TGGAGACAGGGGCCCTTTTGCTTCAGGGTTTCATCCAGGATCGAGCAGGGCGAATGGGGGG
GGAGGCACCCGAGCTGGCCCTGGACCCGGTGCCTCAGGATGCGTCCACCAAGAAGCTGAGC
GAGTGTCTCAAGCGCATCGGGGACGAACTGGACAGTAACATGGAGCTGCAGAGGATGATTG
CCGCCGTGGACACAGACTCCCCCCGAGAGGCCTTTTTCCGAGTGGCAGCTGACATGTTTTCT
GACGGCAACTTCAACTGGGGCCGGGTTGTCGCCCTTTTCTACTTTGCCAGCAAACTGGTGCT
CAAGGCCCTGTGCACCAAGGTGCCGGAACTGATCAGAACCATCATGGGCTGGACATTGGAC
TTCCTCCGGGAGCGGCTGTTGGGCTGGATCCAAGACCAGGGTGGTTGGGACGGCCTCCTCTC
CTACTTTGGGACGCCCACGTGGCAGACCGTGACCATCTTTGTGGCGGGAGTGCTCACCGCCT
CACTCACCATCTGGAAGAAGATGGGCTGA

pBiFC-VN-Bax Asp33Ala

ATGGACTACAAAGACGATGACGACAAGCTTGCGGCCGCGAATTCAATGGTGAGCAAGGGCG
AGGAGCTGTTCACCGGGGTGGTGCCCATCCTGGTCGAGCTGGACGGCGACGTAAACGGCCA
CAAGTTCAGCGTGTCCGGCGAGGGCGAGGGCGATGCCACCTACGGCAAGCTGACCCTGAAG
CTGATCTGCACCACCGGCAAGCTGCCCGTGCCCTGGCCCACCCTCGTGACCACCCTGGGCTA
CGGCCTGCAGTGCTTCGCCCGCTACCCCGACCACATGAAGCAGCACGACTTCTTCAAGTCCG
CCATGCCCGAAGGCTACGTCCAGGAGCGCACCATCTTCTTCAAGGACGACGGCAACTACAA
GACCCGCGCCGAGGTGAAGTTCGAGGGCGACACCCTGGTGAACCGCATCGAGCTGAAGGGC
ATCGACTTCAAGGAGGACGGCAACATCCTGGGGCACAAGCTGGAGTACAACTACAACAGCC
ACAACGTCTATATCACCGCCGACAAGCAGAAGAACGGCATCAAGGCCAACTTCAAGATCCG
CCACAACATCGAGAGATCCATCGCCACCGCTAGCATGGACGGGTCCGGGGAGCAGCCCAGA
GGCGGGGGGCCCACCAGCTCTGAGCAGATCATGAAGACAGGGGCCCTTTTGCTTCAGGGTTT
CATCCAGGCTCGAGCAGGGCGAATGGGGGGGGAGGCACCCGAGCTGGCCCTGGACCCGGTG
CCTCAGGATGCGTCCACCAAGAAGCTGAGCGAGTGTCTCAAGCGCATCGGGGACGAACTGG
ACAGTAACATGGAGCTGCAGAGGATGATTGCCGCCGTGGACACAGACTCCCCCCGAGAGGC
CTTTTTCCGAGTGGCAGCTGACATGTTTTCTGACGGCAACTTCAACTGGGGCCGGGTTGTCGC
CCTTTTCTACTTTGCCAGCAAACTGGTGCTCAAGGCCCTGTGCACCAAGGTGCCGGAACTGA
TCAGAACCATCATGGGCTGGACATTGGACTTCCTCCGGGAGCGGCTGTTGGGCTGGATCCAA
GACCAGGGTGGTTGGGACGGCCTCCTCTCCTACTTTGGGACGCCCACGTGGCAGACCGTGAC
CATCTTTGTGGCGGGAGTGCTCACCGCCTCACTCACCATCTGGAAGAAGATGGGCTGA

pBiFC-VC-Bax Asp33Ala

ATGTACCCATACGATGTTCCAGATTACGCTCTTATGGCCATGGAGGCCCGAATTCGGGACAA
GCAGAAGAACGGCATCAAGGCCAACTTCAAGATCCGCCACAACATCGAGGACGGCGGCGTG
CAGCTCGCCGACCACTACCAGCAGAACACCCCCATCGGCGACGGCCCCGTGCTGCTGCCCGA
CAACCACTACCTGAGCTACCAGTCCAAACTGAGCAAAGACCCCAACGAGAAGCGCGATCAC
ATGGTCCTGCTGGAGTTCGTGACCGCCGCCGGGATCACTCTCGGCATGGACGAGCTGTACAA
GGGTACCCGTCCGGCGTGCAAAATCCCGAACGACCTGAAACAGAAAGTCATGAACCACATC
GATATGGACGGGTCCGGGGAGCAGCCCAGAGGCGGGGGGCCCACCAGCTCTGAGCAGATCA
TGAAGACAGGGGCCCTTTTGCTTCAGGGTTTCATCCAGGCTCGAGCAGGGCGAATGGGGGG
GGAGGCACCCGAGCTGGCCCTGGACCCGGTGCCTCAGGATGCGTCCACCAAGAAGCTGAGC
GAGTGTCTCAAGCGCATCGGGGACGAACTGGACAGTAACATGGAGCTGCAGAGGATGATTG
CCGCCGTGGACACAGACTCCCCCCGAGAGGCCTTTTTCCGAGTGGCAGCTGACATGTTTTCT
GACGGCAACTTCAACTGGGGCCGGGTTGTCGCCCTTTTCTACTTTGCCAGCAAACTGGTGCT
CAAGGCCCTGTGCACCAAGGTGCCGGAACTGATCAGAACCATCATGGGCTGGACATTGGAC
TTCCTCCGGGAGCGGCTGTTGGGCTGGATCCAAGACCAGGGTGGTTGGGACGGCCTCCTCTC
CTACTTTGGGACGCCCACGTGGCAGACCGTGACCATCTTTGTGGCGGGAGTGCTCACCGCCT
CACTCACCATCTGGAAGAAGATGGGCTGA

pBiFC-VN-Bax Trp139Ala

ATGGACTACAAAGACGATGACGACAAGCTTGCGGCCGCGAATTCAATGGTGAGCAAGGGCG
AGGAGCTGTTCACCGGGGTGGTGCCCATCCTGGTCGAGCTGGACGGCGACGTAAACGGCCA
CAAGTTCAGCGTGTCCGGCGAGGGCGAGGGCGATGCCACCTACGGCAAGCTGACCCTGAAG
CTGATCTGCACCACCGGCAAGCTGCCCGTGCCCTGGCCCACCCTCGTGACCACCCTGGGCTA
CGGCCTGCAGTGCTTCGCCCGCTACCCCGACCACATGAAGCAGCACGACTTCTTCAAGTCCG
CCATGCCCGAAGGCTACGTCCAGGAGCGCACCATCTTCTTCAAGGACGACGGCAACTACAA
GACCCGCGCCGAGGTGAAGTTCGAGGGCGACACCCTGGTGAACCGCATCGAGCTGAAGGGC
ATCGACTTCAAGGAGGACGGCAACATCCTGGGGCACAAGCTGGAGTACAACTACAACAGCC
ACAACGTCTATATCACCGCCGACAAGCAGAAGAACGGCATCAAGGCCAACTTCAAGATCCG
CCACAACATCGAGAGATCCATCGCCACCGCTAGCATGGACGGGTCCGGGGAGCAGCCCAGA
GGCGGGGGGCCCACCAGCTCTGAGCAGATCATGAAGACAGGGGCCCTTTTGCTTCAGGGTTT
CATCCAGGATCGAGCAGGGCGAATGGGGGGGGAGGCACCCGAGCTGGCCCTGGACCCGGTG
CCTCAGGATGCGTCCACCAAGAAGCTGAGCGAGTGTCTCAAGCGCATCGGGGACGAACTGG
ACAGTAACATGGAGCTGCAGAGGATGATTGCCGCCGTGGACACAGACTCCCCCCGAGAGGC
CTTTTTCCGAGTGGCAGCTGACATGTTTTCTGACGGCAACTTCAACTGGGGCCGGGTTGTCGC
CCTTTTCTACTTTGCCAGCAAACTGGTGCTCAAGGCCCTGTGCACCAAGGTGCCGGAACTGA
TCAGAACCATCATGGGCGCGACATTGGACTTCCTCCGGGAGCGGCTGTTGGGCTGGATCCAA
GACCAGGGTGGTTGGGACGGCCTCCTCTCCTACTTTGGGACGCCCACGTGGCAGACCGTGAC
CATCTTTGTGGCGGGAGTGCTCACCGCCTCACTCACCATCTGGAAGAAGATGGGCTGA

pBiFC-VC-Bax Trp139Ala

ATGTACCCATACGATGTTCCAGATTACGCTCTTATGGCCATGGAGGCCCGAATTCGGGACAA
GCAGAAGAACGGCATCAAGGCCAACTTCAAGATCCGCCACAACATCGAGGACGGCGGCGTG
CAGCTCGCCGACCACTACCAGCAGAACACCCCCATCGGCGACGGCCCCGTGCTGCTGCCCGA
CAACCACTACCTGAGCTACCAGTCCAAACTGAGCAAAGACCCCAACGAGAAGCGCGATCAC
ATGGTCCTGCTGGAGTTCGTGACCGCCGCCGGGATCACTCTCGGCATGGACGAGCTGTACAA
GGGTACCCGTCCGGCGTGCAAAATCCCGAACGACCTGAAACAGAAAGTCATGAACCACATC
GATATGGACGGGTCCGGGGAGCAGCCCAGAGGCGGGGGGCCCACCAGCTCTGAGCAGATCA
TGAAGACAGGGGCCCTTTTGCTTCAGGGTTTCATCCAGGATCGAGCAGGGCGAATGGGGGG
GGAGGCACCCGAGCTGGCCCTGGACCCGGTGCCTCAGGATGCGTCCACCAAGAAGCTGAGC
GAGTGTCTCAAGCGCATCGGGGACGAACTGGACAGTAACATGGAGCTGCAGAGGATGATTG
CCGCCGTGGACACAGACTCCCCCCGAGAGGCCTTTTTCCGAGTGGCAGCTGACATGTTTTCT
GACGGCAACTTCAACTGGGGCCGGGTTGTCGCCCTTTTCTACTTTGCCAGCAAACTGGTGCT
CAAGGCCCTGTGCACCAAGGTGCCGGAACTGATCAGAACCATCATGGGCGCGACATTGGAC
TTCCTCCGGGAGCGGCTGTTGGGCTGGATCCAAGACCAGGGTGGTTGGGACGGCCTCCTCTC
CTACTTTGGGACGCCCACGTGGCAGACCGTGACCATCTTTGTGGCGGGAGTGCTCACCGCCT
CACTCACCATCTGGAAGAAGATGGGCTGA

pBiFC-VN-Bax Glu146Ala

ATGGACTACAAAGACGATGACGACAAGCTTGCGGCCGCGAATTCAATGGTGAGCAAGGGCG
AGGAGCTGTTCACCGGGGTGGTGCCCATCCTGGTCGAGCTGGACGGCGACGTAAACGGCCA
CAAGTTCAGCGTGTCCGGCGAGGGCGAGGGCGATGCCACCTACGGCAAGCTGACCCTGAAG
CTGATCTGCACCACCGGCAAGCTGCCCGTGCCCTGGCCCACCCTCGTGACCACCCTGGGCTA
CGGCCTGCAGTGCTTCGCCCGCTACCCCGACCACATGAAGCAGCACGACTTCTTCAAGTCCG
CCATGCCCGAAGGCTACGTCCAGGAGCGCACCATCTTCTTCAAGGACGACGGCAACTACAA
GACCCGCGCCGAGGTGAAGTTCGAGGGCGACACCCTGGTGAACCGCATCGAGCTGAAGGGC
ATCGACTTCAAGGAGGACGGCAACATCCTGGGGCACAAGCTGGAGTACAACTACAACAGCC
ACAACGTCTATATCACCGCCGACAAGCAGAAGAACGGCATCAAGGCCAACTTCAAGATCCG
CCACAACATCGAGAGATCCATCGCCACCGCTAGCATGGACGGGTCCGGGGAGCAGCCCAGA
GGCGGGGGGCCCACCAGCTCTGAGCAGATCATGAAGACAGGGGCCCTTTTGCTTCAGGGTTT
CATCCAGGATCGAGCAGGGCGAATGGGGGGGGAGGCACCCGAGCTGGCCCTGGACCCGGTG
CCTCAGGATGCGTCCACCAAGAAGCTGAGCGAGTGTCTCAAGCGCATCGGGGACGAACTGG
ACAGTAACATGGAGCTGCAGAGGATGATTGCCGCCGTGGACACAGACTCCCCCCGAGAGGC
CTTTTTCCGAGTGGCAGCTGACATGTTTTCTGACGGCAACTTCAACTGGGGCCGGGTTGTCGC
CCTTTTCTACTTTGCCAGCAAACTGGTGCTCAAGGCCCTGTGCACCAAGGTGCCGGAACTGA
TCAGAACCATCATGGGCTGGACATTGGACTTCCTCCGGGCGCGGCTGTTGGGCTGGATCCAA
GACCAGGGTGGTTGGGACGGCCTCCTCTCCTACTTTGGGACGCCCACGTGGCAGACCGTGAC
CATCTTTGTGGCGGGAGTGCTCACCGCCTCACTCACCATCTGGAAGAAGATGGGCTGA

pBiFC-VC-Bax Glu146Ala

ATGTACCCATACGATGTTCCAGATTACGCTCTTATGGCCATGGAGGCCCGAATTCGGGACAA
GCAGAAGAACGGCATCAAGGCCAACTTCAAGATCCGCCACAACATCGAGGACGGCGGCGTG
CAGCTCGCCGACCACTACCAGCAGAACACCCCCATCGGCGACGGCCCCGTGCTGCTGCCCGA
CAACCACTACCTGAGCTACCAGTCCAAACTGAGCAAAGACCCCAACGAGAAGCGCGATCAC
ATGGTCCTGCTGGAGTTCGTGACCGCCGCCGGGATCACTCTCGGCATGGACGAGCTGTACAA
GGGTACCCGTCCGGCGTGCAAAATCCCGAACGACCTGAAACAGAAAGTCATGAACCACATC
GATATGGACGGGTCCGGGGAGCAGCCCAGAGGCGGGGGGCCCACCAGCTCTGAGCAGATCA
TGAAGACAGGGGCCCTTTTGCTTCAGGGTTTCATCCAGGATCGAGCAGGGCGAATGGGGGG
GGAGGCACCCGAGCTGGCCCTGGACCCGGTGCCTCAGGATGCGTCCACCAAGAAGCTGAGC
GAGTGTCTCAAGCGCATCGGGGACGAACTGGACAGTAACATGGAGCTGCAGAGGATGATTG
CCGCCGTGGACACAGACTCCCCCCGAGAGGCCTTTTTCCGAGTGGCAGCTGACATGTTTTCT
GACGGCAACTTCAACTGGGGCCGGGTTGTCGCCCTTTTCTACTTTGCCAGCAAACTGGTGCT
CAAGGCCCTGTGCACCAAGGTGCCGGAACTGATCAGAACCATCATGGGCTGGACATTGGAC
TTCCTCCGGGCGCGGCTGTTGGGCTGGATCCAAGACCAGGGTGGTTGGGACGGCCTCCTCTC
CTACTTTGGGACGCCCACGTGGCAGACCGTGACCATCTTTGTGGCGGGAGTGCTCACCGCCT
CACTCACCATCTGGAAGAAGATGGGCTGA

pBiFC-VN-Bax Arg147Ala

ATGGACTACAAAGACGATGACGACAAGCTTGCGGCCGCGAATTCAATGGTGAGCAAGGGCG
AGGAGCTGTTCACCGGGGTGGTGCCCATCCTGGTCGAGCTGGACGGCGACGTAAACGGCCA
CAAGTTCAGCGTGTCCGGCGAGGGCGAGGGCGATGCCACCTACGGCAAGCTGACCCTGAAG
CTGATCTGCACCACCGGCAAGCTGCCCGTGCCCTGGCCCACCCTCGTGACCACCCTGGGCTA
CGGCCTGCAGTGCTTCGCCCGCTACCCCGACCACATGAAGCAGCACGACTTCTTCAAGTCCG
CCATGCCCGAAGGCTACGTCCAGGAGCGCACCATCTTCTTCAAGGACGACGGCAACTACAA
GACCCGCGCCGAGGTGAAGTTCGAGGGCGACACCCTGGTGAACCGCATCGAGCTGAAGGGC
ATCGACTTCAAGGAGGACGGCAACATCCTGGGGCACAAGCTGGAGTACAACTACAACAGCC
ACAACGTCTATATCACCGCCGACAAGCAGAAGAACGGCATCAAGGCCAACTTCAAGATCCG
CCACAACATCGAGAGATCCATCGCCACCGCTAGCATGGACGGGTCCGGGGAGCAGCCCAGA
GGCGGGGGGCCCACCAGCTCTGAGCAGATCATGAAGACAGGGGCCCTTTTGCTTCAGGGTTT
CATCCAGGATCGAGCAGGGCGAATGGGGGGGGAGGCACCCGAGCTGGCCCTGGACCCGGTG
CCTCAGGATGCGTCCACCAAGAAGCTGAGCGAGTGTCTCAAGCGCATCGGGGACGAACTGG
ACAGTAACATGGAGCTGCAGAGGATGATTGCCGCCGTGGACACAGACTCCCCCCGAGAGGC
CTTTTTCCGAGTGGCAGCTGACATGTTTTCTGACGGCAACTTCAACTGGGGCCGGGTTGTCGC
CCTTTTCTACTTTGCCAGCAAACTGGTGCTCAAGGCCCTGTGCACCAAGGTGCCGGAACTGA
TCAGAACCATCATGGGCTGGACATTGGACTTCCTCCGGGAGGCGCTGTTGGGCTGGATCCAA
GACCAGGGTGGTTGGGACGGCCTCCTCTCCTACTTTGGGACGCCCACGTGGCAGACCGTGAC
CATCTTTGTGGCGGGAGTGCTCACCGCCTCACTCACCATCTGGAAGAAGATGGGCTGA

pBiFC-VC-Bax Arg147Ala

ATGTACCCATACGATGTTCCAGATTACGCTCTTATGGCCATGGAGGCCCGAATTCGGGACAA
GCAGAAGAACGGCATCAAGGCCAACTTCAAGATCCGCCACAACATCGAGGACGGCGGCGTG
CAGCTCGCCGACCACTACCAGCAGAACACCCCCATCGGCGACGGCCCCGTGCTGCTGCCCGA
CAACCACTACCTGAGCTACCAGTCCAAACTGAGCAAAGACCCCAACGAGAAGCGCGATCAC
ATGGTCCTGCTGGAGTTCGTGACCGCCGCCGGGATCACTCTCGGCATGGACGAGCTGTACAA
GGGTACCCGTCCGGCGTGCAAAATCCCGAACGACCTGAAACAGAAAGTCATGAACCACATC
GATATGGACGGGTCCGGGGAGCAGCCCAGAGGCGGGGGGCCCACCAGCTCTGAGCAGATCA
TGAAGACAGGGGCCCTTTTGCTTCAGGGTTTCATCCAGGATCGAGCAGGGCGAATGGGGGG
GGAGGCACCCGAGCTGGCCCTGGACCCGGTGCCTCAGGATGCGTCCACCAAGAAGCTGAGC
GAGTGTCTCAAGCGCATCGGGGACGAACTGGACAGTAACATGGAGCTGCAGAGGATGATTG
CCGCCGTGGACACAGACTCCCCCCGAGAGGCCTTTTTCCGAGTGGCAGCTGACATGTTTTCT
GACGGCAACTTCAACTGGGGCCGGGTTGTCGCCCTTTTCTACTTTGCCAGCAAACTGGTGCT
CAAGGCCCTGTGCACCAAGGTGCCGGAACTGATCAGAACCATCATGGGCTGGACATTGGAC
TTCCTCCGGGAGGCGCTGTTGGGCTGGATCCAAGACCAGGGTGGTTGGGACGGCCTCCTCTC
CTACTTTGGGACGCCCACGTGGCAGACCGTGACCATCTTTGTGGCGGGAGTGCTCACCGCCT
CACTCACCATCTGGAAGAAGATGGGCTGA

pBiFC-VN-Bax ΔBH3

ATGGACTACAAAGACGATGACGACAAGCTTGCGGCCGCGAATTCAATGGTGAGCAAGGGCG
AGGAGCTGTTCACCGGGGTGGTGCCCATCCTGGTCGAGCTGGACGGCGACGTAAACGGCCA
CAAGTTCAGCGTGTCCGGCGAGGGCGAGGGCGATGCCACCTACGGCAAGCTGACCCTGAAG
CTGATCTGCACCACCGGCAAGCTGCCCGTGCCCTGGCCCACCCTCGTGACCACCCTGGGCTA
CGGCCTGCAGTGCTTCGCCCGCTACCCCGACCACATGAAGCAGCACGACTTCTTCAAGTCCG
CCATGCCCGAAGGCTACGTCCAGGAGCGCACCATCTTCTTCAAGGACGACGGCAACTACAA
GACCCGCGCCGAGGTGAAGTTCGAGGGCGACACCCTGGTGAACCGCATCGAGCTGAAGGGC
ATCGACTTCAAGGAGGACGGCAACATCCTGGGGCACAAGCTGGAGTACAACTACAACAGCC
ACAACGTCTATATCACCGCCGACAAGCAGAAGAACGGCATCAAGGCCAACTTCAAGATCCG
CCACAACATCGAGAGATCCATCGCCACCGCTAGCATGGACGGGTCCGGGGAGCAGCCCAGA
GGCGGGGGGCCCACCAGCTCTGAGCAGATCATGAAGACAGGGGCCCTTTTGCTTCAGGGTTT
CATCCAGGATCGAGCAGGGCGAATGGGGGGGGAGGCACCCGAGCTGGCCCTGGACCCGGTG
CCTCAGGATGCGTCCACCCAGAGGATGATTGCCGCCGTGGACACAGACTCCCCCCGAGAGG
CCTTTTTCCGAGTGGCAGCTGACATGTTTTCTGACGGCAACTTCAACTGGGGCCGGGTTGTCG
CCCTTTTCTACTTTGCCAGCAAACTGGTGCTCAAGGCCCTGTGCACCAAGGTGCCGGAACTG
ATCAGAACCATCATGGGCTGGACATTGGACTTCCTCCGGGAGCGGCTGTTGGGCTGGATCCA
AGACCAGGGTGGTTGGGACGGCCTCCTCTCCTACTTTGGGACGCCCACGTGGCAGACCGTGA
CCATCTTTGTGGCGGGAGTGCTCACCGCCTCACTCACCATCTGGAAGAAGATGGGCTGA

pBiFC-VC-Bax ΔBH3

ATGTACCCATACGATGTTCCAGATTACGCTCTTATGGCCATGGAGGCCCGAATTCGGGACAA
GCAGAAGAACGGCATCAAGGCCAACTTCAAGATCCGCCACAACATCGAGGACGGCGGCGTG
CAGCTCGCCGACCACTACCAGCAGAACACCCCCATCGGCGACGGCCCCGTGCTGCTGCCCGA
CAACCACTACCTGAGCTACCAGTCCAAACTGAGCAAAGACCCCAACGAGAAGCGCGATCAC
ATGGTCCTGCTGGAGTTCGTGACCGCCGCCGGGATCACTCTCGGCATGGACGAGCTGTACAA
GGGTACCCGTCCGGCGTGCAAAATCCCGAACGACCTGAAACAGAAAGTCATGAACCACATC
GATATGGACGGGTCCGGGGAGCAGCCCAGAGGCGGGGGGCCCACCAGCTCTGAGCAGATCA
TGAAGACAGGGGCCCTTTTGCTTCAGGGTTTCATCCAGGATCGAGCAGGGCGAATGGGGGG
GGAGGCACCCGAGCTGGCCCTGGACCCGGTGCCTCAGGATGCGTCCACCCAGAGGATGATT
GCCGCCGTGGACACAGACTCCCCCCGAGAGGCCTTTTTCCGAGTGGCAGCTGACATGTTTTC
TGACGGCAACTTCAACTGGGGCCGGGTTGTCGCCCTTTTCTACTTTGCCAGCAAACTGGTGCT
CAAGGCCCTGTGCACCAAGGTGCCGGAACTGATCAGAACCATCATGGGCTGGACATTGGAC
TTCCTCCGGGAGCGGCTGTTGGGCTGGATCCAAGACCAGGGTGGTTGGGACGGCCTCCTCTC
CTACTTTGGGACGCCCACGTGGCAGACCGTGACCATCTTTGTGGCGGGAGTGCTCACCGCCT
CACTCACCATCTGGAAGAAGATGGCTGA

pBiFC-VN-Bak

ATGGACTACAAAGACGATGACGACAAGCTTGCGGCCGCGAATTCAATGGTGAGCAAGGGCG
AGGAGCTGTTCACCGGGGTGGTGCCCATCCTGGTCGAGCTGGACGGCGACGTAAACGGCCA
CAAGTTCAGCGTGTCCGGCGAGGGCGAGGGCGATGCCACCTACGGCAAGCTGACCCTGAAG
CTGATCTGCACCACCGGCAAGCTGCCCGTGCCCTGGCCCACCCTCGTGACCACCCTGGGCTA
CGGCCTGCAGTGCTTCGCCCGCTACCCCGACCACATGAAGCAGCACGACTTCTTCAAGTCCG
CCATGCCCGAAGGCTACGTCCAGGAGCGCACCATCTTCTTCAAGGACGACGGCAACTACAA
GACCCGCGCCGAGGTGAAGTTCGAGGGCGACACCCTGGTGAACCGCATCGAGCTGAAGGGC
ATCGACTTCAAGGAGGACGGCAACATCCTGGGGCACAAGCTGGAGTACAACTACAACAGCC
ACAACGTCTATATCACCGCCGACAAGCAGAAGAACGGCATCAAGGCCAACTTCAAGATCCG
CCACAACATCGAGAGATCCATCGCCACCGCTAGCATGGCTTCGGGGCAAGGCCCAGGTCCTC
CCAGGCAGGAGTGCGGAGAGCCTGCCCTGCCCTCTGCTTCTGAGGAGCAGGTAGCCCAGGA
CACAGAGGAGGTTTTCCGCAGCTACGTTTTTTACCGCCATCAGCAGGAACAGGAGGCTGAAG
GGGTGGCTGCCCCTGCCGACCCAGAGATGGTCACCTTACCTCTGCAACCTAGCAGCACCATG
GGGCAGGTGGGACGGCAGCTCGCCATCATCGGGGACGACATCAACCGACGCTATGACTCAG
AGTTCCAGACCATGTTGCAGCACCTGCAGCCCACGGCAGAGAATGCCTATGAGTACTTCACC
AAGATTGCCACCAGCCTGTTTGAGAGTGGCATCAATTGGGGCCGTGTGGTGGCTCTTCTGGG
CTTCGGCTACCGTCTGGCCCTACACGTCTACCAGCATGGCCTGACTGGCTTCCTAGGCCAGG
TGACCCGCTTCGTGGTCGACTTCATGCTGCATCACTGCATTGCCCGGTGGATTGCACAGAGG
GGTGGCTGGGTGGCAGCCCTGAACTTGGGCAATGGTCCCATCCTGAACGTGCTGGTGGTTCT
GGGTGTGGTTCTGTTGGGCCAGTTTGTGGTACGAAGATTCTTCAAATCATGA

pBiFC-VC-Bak

ATGTACCCATACGATGTTCCAGATTACGCTCTTATGGCCATGGAGGCCCGAATTCGGGACAA
GCAGAAGAACGGCATCAAGGCCAACTTCAAGATCCGCCACAACATCGAGGACGGCGGCGTG
CAGCTCGCCGACCACTACCAGCAGAACACCCCCATCGGCGACGGCCCCGTGCTGCTGCCCGA
CAACCACTACCTGAGCTACCAGTCCGCCCTGAGCAAAGACCCCAACGAGAAGCGCGATCAC
ATGGTCCTGCTGGAGTTCGTGACCGCCGCCGGGATCACTCTCGGCATGGACGAGCTGTACAA
GGGTACCCGTCCGGCGTGCAAAATCCCGAACGACCTGAAACAGAAAGTCATGAACCACATC
GATATGGCTTCGGGGCAAGGCCCAGGTCCTCCCAGGCAGGAGTGCGGAGAGCCTGCCCTGC
CCTCTGCTTCTGAGGAGCAGGTAGCCCAGGACACAGAGGAGGTTTTCCGCAGCTACGTTTTT
TACCGCCATCAGCAGGAACAGGAGGCTGAAGGGGTGGCTGCCCCTGCCGACCCAGAGATGG
TCACCTTACCTCTGCAACCTAGCAGCACCATGGGGCAGGTGGGACGGCAGCTCGCCATCATC
GGGGACGACATCAACCGACGCTATGACTCAGAGTTCCAGACCATGTTGCAGCACCTGCAGC
CCACGGCAGAGAATGCCTATGAGTACTTCACCAAGATTGCCACCAGCCTGTTTGAGAGTGGC
ATCAATTGGGGCCGTGTGGTGGCTCTTCTGGGCTTCGGCTACCGTCTGGCCCTACACGTCTAC
CAGCATGGCCTGACTGGCTTCCTAGGCCAGGTGACCCGCTTCGTGGTCGACTTCATGCTGCA
TCACTGCATTGCCCGGTGGATTGCACAGAGGGGTGGCTGGGTGGCAGCCCTGAACTTGGGCA
ATGGTCCCATCCTGAACGTGCTGGTGGTTCTGGGTGTGGTTCTGTTGGGCCAGTTTGTGGTAC
GAAGATTCTTCAAATCATGA

pBiFC-VN-Bak ΔH1α

ATGGACTACAAAGACGATGACGACAAGCTTGCGGCCGCGAATTCAATGGTGAGCAAGGGCG
AGGAGCTGTTCACCGGGGTGGTGCCCATCCTGGTCGAGCTGGACGGCGACGTAAACGGCCA
CAAGTTCAGCGTGTCCGGCGAGGGCGAGGGCGATGCCACCTACGGCAAGCTGACCCTGAAG
CTGATCTGCACCACCGGCAAGCTGCCCGTGCCCTGGCCCACCCTCGTGACCACCCTGGGCTA
CGGCCTGCAGTGCTTCGCCCGCTACCCCGACCACATGAAGCAGCACGACTTCTTCAAGTCCG
CCATGCCCGAAGGCTACGTCCAGGAGCGCACCATCTTCTTCAAGGACGACGGCAACTACAA
GACCCGCGCCGAGGTGAAGTTCGAGGGCGACACCCTGGTGAACCGCATCGAGCTGAAGGGC
ATCGACTTCAAGGAGGACGGCAACATCCTGGGGCACAAGCTGGAGTACAACTACAACAGCC
ACAACGTCTATATCACCGCCGACAAGCAGAAGAACGGCATCAAGGCCAACTTCAAGATCCG
CCACAACATCGAGAGATCCATCGCCACCGCTAGCATGGCTTCGGGGCAAGGCCCAGGTCCTC
CCAGGCAGGAGTGCGGAGAGCCTGCCCTGCCCTCTGCTTCTGAAGGGGTGGCTGCCCCTGCC
GACCCAGAGATGGTCACCTTACCTCTGCAACCTAGCAGCACCATGGGGCAGGTGGGACGGC
AGCTCGCCATCATCGGGGACGACATCAACCGACGCTATGACTCAGAGTTCCAGACCATGTTG
CAGCACCTGCAGCCCACGGCAGAGAATGCCTATGAGTACTTCACCAAGATTGCCACCAGCCT
GTTTGAGAGTGGCATCAATTGGGGCCGTGTGGTGGCTCTTCTGGGCTTCGGCTACCGTCTGG
CCCTACACGTCTACCAGCATGGCCTGACTGGCTTCCTAGGCCAGGTGACCCGCTTCGTGGTC
GACTTCATGCTGCATCACTGCATTGCCCGGTGGATTGCACAGAGGGGTGGCTGGGTGGCAGC
CCTGAACTTGGGCAATGGTCCCATCCTGAACGTGCTGGTGGTTCTGGGTGTGGTTCTGTTGG
GCCAGTTTGTGGTACGAAGATTCTTCAAATCATGA

pBiFC-VC-Bak ΔH1α

ATGTACCCATACGATGTTCCAGATTACGCTCTTATGGCCATGGAGGCCCGAATTCGGGACAA
GCAGAAGAACGGCATCAAGGCCAACTTCAAGATCCGCCACAACATCGAGGACGGCGGCGTG
CAGCTCGCCGACCACTACCAGCAGAACACCCCCATCGGCGACGGCCCCGTGCTGCTGCCCGA
CAACCACTACCTGAGCTACCAGTCCGCCCTGAGCAAAGACCCCAACGAGAAGCGCGATCAC
ATGGTCCTGCTGGAGTTCGTGACCGCCGCCGGGATCACTCTCGGCATGGACGAGCTGTACAA
GGGTACCCGTCCGGCGTGCAAAATCCCGAACGACCTGAAACAGAAAGTCATGAACCACATC
GATATGGCTTCGGGGCAAGGCCCAGGTCCTCCCAGGCAGGAGTGCGGAGAGCCTGCCCTGC
CCTCTGCTTCTGAAGGGGTGGCTGCCCCTGCCGACCCAGAGATGGTCACCTTACCTCTGCAA
CCTAGCAGCACCATGGGGCAGGTGGGACGGCAGCTCGCCATCATCGGGGACGACATCAACC
GACGCTATGACTCAGAGTTCCAGACCATGTTGCAGCACCTGCAGCCCACGGCAGAGAATGC
CTATGAGTACTTCACCAAGATTGCCACCAGCCTGTTTGAGAGTGGCATCAATTGGGGCCGTG
TGGTGGCTCTTCTGGGCTTCGGCTACCGTCTGGCCCTACACGTCTACCAGCATGGCCTGACTG
GCTTCCTAGGCCAGGTGACCCGCTTCGTGGTCGACTTCATGCTGCATCACTGCATTGCCCGGT
GGATTGCACAGAGGGGTGGCTGGGTGGCAGCCCTGAACTTGGGCAATGGTCCCATCCTGAA
CGTGCTGGTGGTTCTGGGTGTGGTTCTGTTGGGCCAGTTTGTGGTACGAAGATTCTTCAAATC
ATGA

pBiFC-VN-Bak His164Ala

ATGGACTACAAAGACGATGACGACAAGCTTGCGGCCGCGAATTCAATGGTGAGCAAGGGCG
AGGAGCTGTTCACCGGGGTGGTGCCCATCCTGGTCGAGCTGGACGGCGACGTAAACGGCCA
CAAGTTCAGCGTGTCCGGCGAGGGCGAGGGCGATGCCACCTACGGCAAGCTGACCCTGAAG
CTGATCTGCACCACCGGCAAGCTGCCCGTGCCCTGGCCCACCCTCGTGACCACCCTGGGCTA
CGGCCTGCAGTGCTTCGCCCGCTACCCCGACCACATGAAGCAGCACGACTTCTTCAAGTCCG
CCATGCCCGAAGGCTACGTCCAGGAGCGCACCATCTTCTTCAAGGACGACGGCAACTACAA
GACCCGCGCCGAGGTGAAGTTCGAGGGCGACACCCTGGTGAACCGCATCGAGCTGAAGGGC
ATCGACTTCAAGGAGGACGGCAACATCCTGGGGCACAAGCTGGAGTACAACTACAACAGCC
ACAACGTCTATATCACCGCCGACAAGCAGAAGAACGGCATCAAGGCCAACTTCAAGATCCG
CCACAACATCGAGAGATCCATCGCCACCGCTAGCATGGCTTCGGGGCAAGGCCCAGGTCCTC
CCAGGCAGGAGTGCGGAGAGCCTGCCCTGCCCTCTGCTTCTGAGGAGCAGGTAGCCCAGGA
CACAGAGGAGGTTTTCCGCAGCTACGTTTTTACCGCCATCAGCAGGAACAGGAGGCTGAAG
GGGTGGCTGCCCCTGCCGACCCAGAGATGGTCACCTTACCTCTGCAACCTAGCAGCACCATG
GGGCAGGTGGGACGGCAGCTCGCCATCATCGGGGACGACATCAACCGACGCTATGACTCAG
AGTTCCAGACCATGTTGCAGCACCTGCAGCCCACGGCAGAGAATGCCTATGAGTACTTCACC
AAGATTGCCACCAGCCTGTTTGAGAGTGGCATCAATTGGGGCCGTGTGGTGGCTCTTCTGGG
CTTCGGCTACCGTCTGGCCCTACACGTCTACCAGCATGGCCTGACTGGCTTCCTAGGCCAGG
TGACCCGCTTCGTGGTCGACTTCATGCTGGCTCACTGCATTGCCCGGTGGATTGCACAGAGG
GGTGGCTGGGTGGCAGCCCTGAACTTGGGCAATGGTCCCATCCTGAACGTGCTGGTGGTTCT
GGGTGTGGTTCTGTTGGGCCAGTTTGTGGTACGAAGATTCTTCAAATCATGA

pBiFC-VC-Bak His164Ala

ATGTACCCATACGATGTTCCAGATTACGCTCTTATGGCCATGGAGGCCCGAATTCGGGACAA
GCAGAAGAACGGCATCAAGGCCAACTTCAAGATCCGCCACAACATCGAGGACGGCGGCGTG
CAGCTCGCCGACCACTACCAGCAGAACACCCCCATCGGCGACGGCCCCGTGCTGCTGCCCGA
CAACCACTACCTGAGCTACCAGTCCGCCCTGAGCAAAGACCCCAACGAGAAGCGCGATCAC
ATGGTCCTGCTGGAGTTCGTGACCGCCGCCGGGATCACTCTCGGCATGGACGAGCTGTACAA
GGGTACCCGTCCGGCGTGCAAAATCCCGAACGACCTGAAACAGAAAGTCATGAACCACATC
GATATGGCTTCGGGGCAAGGCCCAGGTCCTCCCAGGCAGGAGTGCGGAGAGCCTGCCCTGC
CCTCTGCTTCTGAGGAGCAGGTAGCCCAGGACACAGAGGAGGTTTTCCGCAGCTACGTTTTT
TACCGCCATCAGCAGGAACAGGAGGCTGAAGGGGTGGCTGCCCCTGCCGACCCAGAGATGG
TCACCTTACCTCTGCAACCTAGCAGCACCATGGGGCAGGTGGGACGGCAGCTCGCCATCATC
GGGGACGACATCAACCGACGCTATGACTCAGAGTTCCAGACCATGTTGCAGCACCTGCAGC
CCACGGCAGAGAATGCCTATGAGTACTTCACCAAGATTGCCACCAGCCTGTTTGAGAGTGGC
ATCAATTGGGGCCGTGTGGTGGCTCTTCTGGGCTTCGGCTACCGTCTGGCCCTACACGTCTAC
CAGCATGGCCTGACTGGCTTCCTAGGCCAGGTGACCCGCTTCGTGGTCGACTTCATGCTGGC
TCACTGCATTGCCCGGTGGATTGCACAGAGGGGTGGCTGGGTGGCAGCCCTGAACTTGGGCA
ATGGTCCCATCCTGAACGTGCTGGTGGTTCTGGGTGTGGTTCTGTTGGGCCAGTTTGTGGTAC
GAAGATTCTTCAAATCATAG

pBiFC-VN-Bak ΔBH3

ATGGACTACAAAGACGATGACGACAAGCTTGCGGCCGCGAATTCAATGGTGAGCAAGGGCG
AGGAGCTGTTCACCGGGGTGGTGCCCATCCTGGTCGAGCTGGACGGCGACGTAAACGGCCA
CAAGTTCAGCGTGTCCGGCGAGGGCGAGGGCGATGCCACCTACGGCAAGCTGACCCTGAAG
CTGATCTGCACCACCGGCAAGCTGCCCGTGCCCTGGCCCACCCTCGTGACCACCCTGGGCTA
CGGCCTGCAGTGCTTCGCCCGCTACCCCGACCACATGAAGCAGCACGACTTCTTCAAGTCCG
CCATGCCCGAAGGCTACGTCCAGGAGCGCACCATCTTCTTCAAGGACGACGGCAACTACAA
GACCCGCGCCGAGGTGAAGTTCGAGGGCGACACCCTGGTGAACCGCATCGAGCTGAAGGGC
ATCGACTTCAAGGAGGACGGCAACATCCTGGGGCACAAGCTGGAGTACAACTACAACAGCC
ACAACGTCTATATCACCGCCGACAAGCAGAAGAACGGCATCAAGGCCAACTTCAAGATCCG
CCACAACATCGAGAGATCCATCGCCACCGCTAGCATGGCTTCGGGGCAAGGCCCAGGTCCTC
CCAGGCAGGAGTGCGGAGAGCCTGCCCTGCCCTCTGCTTCTGAGGAGCAGGTAGCCCAGGA
CACAGAGGAGGTTTTCCGCAGCTACGTTTTTTACCGCCATCAGCAGGAACAGGAGGCTGAAG
GGGTGGCTGCCCCTGCCGACCCAGAGATGGTCACCTTACCTCTGCAACCTAGCAGCACCATG
GGGCAGTATGACTCAGAGTTCCAGACCATGTTGCAGCACCTGCAGCCCACGGCAGAGAATG
CCTATGAGTACTTCACCAAGATTGCCACCAGCCTGTTTGAGAGTGGCATCAATTGGGGCCGT
GTGGTGGCTCTTCTGGGCTTCGGCTACCGTCTGGCCCTACACGTCTACCAGCATGGCCTGACT
GGCTTCCTAGGCCAGGTGACCCGCTTCGTGGTCGACTTCATGCTGCATCACTGCATTGCCCG
GTGGATTGCACAGAGGGGTGGCTGGGTGGCAGCCCTGAACTTGGGCAATGGTCCCATCCTG
AACGTGCTGGTGGTTCTGGGTGTGGTTCTGTTGGGCCAGTTTGTGGTACGAAGATTCTTCAA
ATCATGA

pBiFC-VC-Bak ΔBH3

ATGTACCCATACGATGTTCCAGATTACGCTCTTATGGCCATGGAGGCCCGAATTCGGGACAA
GCAGAAGAACGGCATCAAGGCCAACTTCAAGATCCGCCACAACATCGAGGACGGCGGCGTG
CAGCTCGCCGACCACTACCAGCAGAACACCCCCATCGGCGACGGCCCCGTGCTGCTGCCCGA
CAACCACTACCTGAGCTACCAGTCCAAACTGAGCAAAGACCCCAACGAGAAGCGCGATCAC
ATGGTCCTGCTGGAGTTCGTGACCGCCGCCGGGATCACTCTCGGCATGGACGAGCTGTACAA
GGGTACCCGTCCGGCGTGCAAAATCCCGAACGACCTGAAACAGAAAGTCATGAACCACATC
GATATGGCTTCGGGGCAAGGCCCAGGTCCTCCCAGGCAGGAGTGCGGAGAGCCTGCCCTGC
CCTCTGCTTCTGAGGAGCAGGTAGCCCAGGACACAGAGGAGGTTTTCCGCAGCTACGTTTTT
TACCGCCATCAGCAGGAACAGGAGGCTGAAGGGGTGGCTGCCCCTGCCGACCCAGAGATGG
TCACCTTACCTCTGCAACCTAGCAGCACCATGGGGCAGTATGACTCAGAGTTCCAGACCATG
TTGCAGCACCTGCAGCCCACGGCAGAGAATGCCTATGAGTACTTCACCAAGATTGCCACCAG
CCTGTTTGAGAGTGGCATCAATTGGGGCCGTGTGGTGGCTCTTCTGGGCTTCGGCTACCGTCT
GGCCCTACACGTCTACCAGCATGGCCTGACTGGCTTCCTAGGCCAGGTGACCCGCTTCGTGG
TCGACTTCATGCTGCATCACTGCATTGCCCGGTGGATTGCACAGAGGGGTGGCTGGGTGGCA
GCCCTGAACTTGGGCAATGGTCCCATCCTGAACGTGCTGGTGGTTCTGGGTGTGGTTCTGTTG
GGCCAGTTTGTGGTACGAAGATTCTTCAAATCATGA

pBiFC-Bim-CC

ATGTACCCATACGATGTTCCAGATTACGCTCTTATGGCCATGGAGGCCATGGCAAAGCAACC
TTCTGATGTAAGTTCTGAGTGTGACCGAGAAGGTAGACAATTGCAGCCTGCGGAGAGGCCTC
CCCAGCTCAGACCTGGGGCCCCTACCTCCCTACAGACAGAGCCACAAGGTAATCCTGAAGG
CAATCACGGAGGTGAAGGGGACAGCTGCCCCCACGGCAGCCCTCAGGGCCCGCTGGCCCCA
CCTGCCAGCCCTGGCCCTTTTGCTACCAGATCCCCGCTTTTCATCTTTATGAGAAGATCCTCC
CTGCTGTCTCGATCCTCCAGTGGGTATTTCTCTTTTGACACAGACAGGAGCCCAGCACCCATG
AGTTGTGACAAATCAACACAAACCCCAAGTCCTCCTTGCCAGGCCTTCAACCACTATCTCAG
TGCAATGGCTTCCATGAGGCAGGCTGAACCTGCAGATATGCGCCCAGAGATATGGATCGCCC
AAGAGTTGCGGCGTATCGGAGACGAGTTTAACGCTTACTATGCAAGGAGGGTATTTTTGAAT
AATTACCAAGCAGCCGAAGACCACCCACGAATGGTTATCTTACGACTGTTACGTTACATTGT
CCGCCTGGTGTGGAGAATGCATGGTACCCGTCCGGCGTGCAAAATCCCGAACGACCTGAAA
CAGAAAGTCATGAACCACGACAAGCAGAAGAACGGCATCAAGGCCAACTTCAAGATCCGCC
ACAACATCGAGGACGGC**AGC**GTGCAGCTCGCCGACCACTACCAGCAGAACACCCCCATCGG
CGACGGCCCCGTGCTGCTGCCCGACAACCACTACCTGAGC**ACC**CAGTCCAAACTGAGCAAA
GACCCCAACGAGAAGCGCGATCACATGGTCCTGCTGGAGTTCGTGACCGCCGCCGGGATCA
CTCTCGGCATGGACGAGCTGTACAAGTGA

pBiFC-Puma-CC

ATGTACCCATACGATGTTCCAGATTACGCTCTTATGGCCATGGAGGCCATGGCCCGCGCACG
CCAGGAGGGCAGCTCCCCGGAGCCCGTAGAGGGCCTGGCCCGCGACGGCCCGCGCCCCTTC
CCGCTCGGCCGCCTGGTGCCCTCGGCAGTGTCCTGCGGCCTCTGCGAGCCCGGCCTGGCTGC
CGCCCCCGCCGCCCCCACCCTGCTGCCCGCTGCCTACCTCTGCGCCCCCACCGCCCCACCCGC
CGTCACCGCCGCCCTGGGGGGTTCCCGCTGGCCTGGGGGTCCCCGCAGCCGGCCCCGAGGCC
CGCGCCCGGACGGTCCTCAGCCCTCGCTCTCGCTGGCGGAGCAGCACCTGGAGTCGCCCGTG
CCCAGCGCCCCGGGGGCTCTGGCGGGCGGTCCCACCCAGGCGGCCCCGGGAGTCCGCGGGG
AGGAGGAACAGTGGGCCCGGGAGATCGGGGCCCAGCTGCGGCGGATGGCGGACGACCTCA
ACGCACAGTACGAGCGGCGGAGACAAGAGGAGCAGCAGCGGCACCGCCCCTCACCCTGGA
GGGTCCTGTACAATCTCATCATGGGACTCCTGCCCTTACCCAGGGGCCACAGAGCCCCCGAG
ATGGAGCCCAATGGTACCCGTCCGGCGTGCAAAATCCCGAACGACCTGAAACAGAAAGTCA
TGAACCACGACAAGCAGAAGAACGGCATCAAGGCCAACTTCAAGATCCGCCACAACATCGA
GGACGGC**AGC**GTGCAGCTCGCCGACCACTACCAGCAGAACACCCCCATCGGCGACGGCCCC
GTGCTGCTGCCCGACAACCACTACCTGAGC**ACC**CAGTCCAAACTGAGCAAAGACCCCAACG
AGAAGCGCGATCACATGGTCCTGCTGGAGTTCGTGACCGCCGCCGGGATCACTCTCGGCATG
GACGAGCTGTACAAGTGA

pBiFC-Noxa-CC

ATGTACCCATACGATGTTCCAGATTACGCTCTTATGGCCATGGAGGCCATGCCTGGGAAGAA
GGCGCGCAAGAACGCTCAACCGAGCCCCGCGCGGGCTCCAGCAGAGCTGGAAGTCGAGTGT
GCTACTCAACTCAGGAGATTTGGAGACAAACTGAACTTCCGGCAGAAACTTCTGAATCTGAT
ATCCAAACTCTTCTGCTCAGGAACCGGTACCCGTCCGGCGTGCAAAATCCCGAACGACCTGA
AACAGAAAGTCATGAACCACGACAAGCAGAAGAACGGCATCAAGGCCAACTTCAAGATCCG
CCACAACATCGAGGACGGC**AGC**GTGCAGCTCGCCGACCACTACCAGCAGAACACCCCCATC
GGCGACGGCCCCGTGCTGCTGCCCGACAACCACTACCTGAGC**ACC**CAGTCCAAACTGAGCA
AAGACCCCAACGAGAAGCGCGATCACATGA

pBiFC-Bax-VN

ATGGACTACAAAGACGATGACGACAAGCTTGCGGCCGCGAATTCAATGGACGGGTCCGGGG
AGCAGCCCAGAGGCGGGGGGCCCACCAGCTCTGAGCAGATCATGAAGACAGGGGCCCTTTT
GCTTCAGGGTTTCATCCAGGATCGAGCAGGGCGAATGGGGGGGGAGGCACCCGAGCTGGCC
CTGGACCCGGTGCCTCAGGATGCGTCCACCAAGAAGCTGAGCGAGTGTCTCAAGCGCATCG
GGGACGAACTGGACAGTAACATGGAGCTGCAGAGGATGATTGCCGCCGTGGACACAGACTC
CCCCCGAGAGGCCTTTTTCCGAGTGGCAGCTGACATGTTTTCTGACGGCAACTTCAACTGGG
GCCGGGTTGTCGCCCTTTTCTACTTTGCCAGCAAACTGGTGCTCAAGGCCCTGTGCACCAAG
GTGCCGGAACTGATCAGAACCATCATGGGCTGGACATTGGACTTCCTCCGGGAGCGGCTGTT
GGGCTGGATCCAAGACCAGGGTGGTTGGGACGGCCTCCTCTCCTACTTTGGGACGCCCACGT
GGCAGACCGTGACCATCTTTGTGGCGGGAGTGCTCACCGCCTCACTCACCATCTGGAAGAAG
ATGGGCGCTAGCAGATCCATCGCCACCATGGTGAGCAAGGGCGAGGAGCTGTTCACCGGGG
TGGTGCCCATCCTGGTCGAGCTGGACGGCGACGTAAACGGCCACAAGTTCAGCGTGTCCGGC
GAGGGCGAGGGCGATGCCACCTACGGCAAGCTGACCCTGAAGCTGATCTGCACCACCGGCA
AGCTGCCCGTGCCCTGGCCCACCCTCGTGACCACCCTGGGCTACGGCCTGCAGTGCTTCGCC
CGCTACCCCGACCACATGAAGCAGCACGACTTCTTCAAGTCCGCCATGCCCGAAGGCTACGT
CCAGGAGCGCACCATCTTCTTCAAGGACGACGGCAACTACAAGACCCGCGCCGAGGTGAAG
TTCGAGGGCGACACCCTGGTGAACCGCATCGAGCTGAAGGGCATCGACTTCAAGGAGGACG
GCAACATCCTGGGGCACAAGCTGGAGTACAACTACAACAGCCACAACGTCTATATCACCGC
CGACAAGCAGAAGAACGGCATCAAGGCCAACTTCAAGATCCGCCACAACATCGAGTGA

pBiFC-Bak-VN

ATGGACTACAAAGACGATGACGACAAGCTTGCGGCCGCGAATTCAATGGCTTCGGGGCAAG
GCCCAGGTCCTCCCAGGCAGGAGTGCGGAGAGCCTGCCCTGCCCTCTGCTTCTGAGGAGCAG
GTAGCCCAGGACACAGAGGAGGTTTTCCGCAGCTACGTTTTTTACCGCCATCAGCAGGAACA
GGAGGCTGAAGGGGTGGCTGCCCCTGCCGACCCAGAGATGGTCACCTTACCTCTGCAACCTA
GCAGCACCATGGGGCAGGTGGGACGGCAGCTCGCCATCATCGGGGACGACATCAACCGACG
CTATGACTCAGAGTTCCAGACCATGTTGCAGCACCTGCAGCCCACGGCAGAGAATGCCTATG
AGTACTTCACCAAGATTGCCACCAGCCTGTTTGAGAGTGGCATCAATTGGGGCCGTGTGGTG
GCTCTTCTGGGCTTCGGCTACCGTCTGGCCCTACACGTCTACCAGCATGGCCTGACTGGCTTC
CTAGGCCAGGTGACCCGCTTCGTGGTCGACTTCATGCTGCATCACTGCATTGCCCGGTGGAT
TGCACAGAGGGGTGGCTGGGTGGCAGCCCTGAACTTGGGCAATGGTCCCATCCTGAACGTG
CTGGTGGTTCTGGGTGTGGTTCTGTTGGGCCAGTTTGTGGTACGAAGATTCTTCAAATCAGCT
AGCAGATCCATCGCCACCATGGTGAGCAAGGGCGAGGAGCTGTTCACCGGGGTGGTGCCCA
TCCTGGTCGAGCTGGACGGCGACGTAAACGGCCACAAGTTCAGCGTGTCCGGCGAGGGCGA
GGGCGATGCCACCTACGGCAAGCTGACCCTGAAGCTGATCTGCACCACCGGCAAGCTGCCC
GTGCCCTGGCCCACCCTCGTGACCACCCTGGGCTACGGCCTGCAGTGCTTCGCCCGCTACCC
CGACCACATGAAGCAGCACGACTTCTTCAAGTCCGCCATGCCCGAAGGCTACGTCCAGGAG
CGCACCATCTTCTTCAAGGACGACGGCAACTACAAGACCCGCGCCGAGGTGAAGTTCGAGG
GCGACACCCTGGTGAACCGCATCGAGCTGAAGGGCATCGACTTCAAGGAGGACGGCAACAT
CCTGGGGCACAAGCTGGAGTACAACTACAACAGCCACAACGTCTATATCACCGCCGACAAG
CAGAAGAACGGCATCAAGGCCAACTTCAAGATCCGCCACAACATCGAGTGA

pBiFC-Mcl-1-CN

ATGGACTACAAAGACGATGACGACAAGCTTGCGGCCGCGAATTCAATGTTTGGCCTCAAAA
GAAACGCGGTAATCGGACTCAACCTCTACTGTGGGGGGGCCGGCTTGGGGGCCGGCAGCGG
CGGCGCCACCCGCCCGGGAGGGCGACTTTTGGCTACGGAGAAGGAGGCCTCGGCCCGGCGA
GAGATAGGGGGAGGGGAGGCCGGCGCGGTGATTGGCGGAAGCGCCGGCGCAAGCCCCCCG
TCCACCCTCACGCCAGACTCCCGGAGGGTCGCGCGGCCGCCGCCCATTGGCGCCGAGGTCCC
CGACGTCACCGCGACCCCCGCGAGGCTGCTTTTCTTCGCGCCCACCCGCCGCGCGGCGCCGC
TTGAGGAGATGGAAGCCCCGGCCGCTGACGCCATCATGTCGCCCGAAGAGGAGCTGGACGG
GTACGAGCCGGAGCCTCTCGGGAAGCGGCCGGCTGTCCTGCCGCTGCTGGAGTTGGTCGGG
GAATCTGGTAATAACACCAGTACGGACGGGTCACTACCCTCGACGCCGCCGCCAGCAGAGG
AGGAGGAGGACGAGTTGTACCGGCAGTCGCTGGAGATTATCTCTCGGTACCTTCGGGAGCA
GGCCACCGGCGCCAAGGACACAAAGCCAATGGGCAGGTCTGGGGCCACCAGCAGGAAGGC
GCTGGAGACCTTACGACGGGTTGGGGATGGCGTGCAGCGCAACCACGAGACGGCCTTCCAA
GGCATGCTTCGGAAACTGGACATCAAAAACGAAGACGATGTGAAATCGTTGTCTCGAGTGA
TGATCCATGTTTTCAGCGACGGCGTAACAAACTGGGGCAGGATTGTGACTCTCATTTCTTTTG
GTGCCTTTGTGGCTAAACACTTGAAGACCATAAACCAAGAAAGCTGCATCGAACCATTAGCA
GAAAGTATCACAGACGTTCTCGTAAGGACAAAACGGGACTGGCTAGTTAAACAAAGAGGCT
GGGATGGGTTTGTGGAGTTCTTCCATGTAGAGGACCTAGAAGGTGGCATCAGGAATGTGCTG
CTGGCTTTTGCAGGTGTTGCTGGAGTAGGAGCTGGTTTGGCATATCTAATAAGAGCTAGCAG
ATCCATCGCCACCATGGTGAGCAAGGGCGAGGAGCTGTTCACCGGGGTGGTGCCCATCCTG
GTCGAGCTGGACGGCGACGTAAACGGCCACAAGTTCAGCGTGTCCGGCGAGGGCGAGGGCG
ATGCCACCTACGGCAAGCTGACCCTGAAGCTGATCTGCACCACCGGCAAGCTGCCCGTGCCC
TGGCCCACCCTCGTGACCACCCTGACCTGGGGCCTGCAGTGCTTCGCCCGCTACCCCGACCA
CATGAAGCAGCACGACTTCTTCAAGTCCGCCATGCCCGAAGGCTACGTCCAGGAGCGCACC
ATCTTCTTCAAGGACGACGGCAACTACAAGACCCGCGCCGAGGTGAAGTTCGAGGGCGACA
CCCTGGTGAACCGCATCGAGCTGAAGGGCATCGACTTCAAGGAGGACGGCAACATCCTGGG
GCACAAGCTGGAGTACAACGCCATCAGCGACAACGTCTATATCACCGCCGACAAGCAGAAG
AACGGCATCAAGGCCAACTTCAAGATCCGCCACAACATCGAGTGA

pBiFC-Bcl-X_L-CN

ATGGACTACAAAGACGATGACGACAAGCTTGCGGCCGCGAATTCAATGTCTCAGAGCAACC
GGGAGCTGGTGGTTGACTTTCTCTCCTACAAGCTTTCCCAGAAAGGATACAGCTGGAGTCAG
TTTAGTGATGTGGAAGAGAACAGGACTGAGGCCCCAGAAGGGACTGAATCGGAGATGGAGA
CCCCCAGTGCCATCAATGGCAACCCATCCTGGCACCTGGCAGACAGCCCCGCGGTGAATGG
AGCCACTGGCCACAGCAGCAGTTTGGATGCCCGGGAGGTGATCCCCATGGCAGCAGTAAAG
CAAGCGCTGAGGGAGGCAGGCGACGAGTTTGAACTGCGGTACCGGCGGGCATTCAGTGACC
TGACATCCCAGCTCCACATCACCCCAGGGACAGCATATCAGAGCTTTGAACAGGTAGTGAAT
GAACTCTTCCGGGATGGGGTAAACTGGGGTCGCATTGTGGCCTTTTTCTCCTTCGGCGGGGC
ACTGTGCGTGGAAAGCGTAGACAAGGAGATGCAGGTATTGGTGAGTCGGATCGCAGCTTGG
ATGGCCACTTACCTGAATGACCACCTAGAGCCTTGGATCCAGGAGAACGGCGGCTGGGATA
CTTTTGTGGAACTCTATGGGAACAATGCAGCAGCCGAGAGCCGAAAGGGCCAGGAACGCTT
CAACCGCTGGTTCCTGACGGGCATGACTGTGGCCGGCGTGGTTCTGCTGGGCTCACTCTTCA
GTCGGAAAGCTAGCAGATCCATCGCCACCATGGTGAGCAAGGGCGAGGAGCTGTTCACCGG
GGTGGTGCCCATCCTGGTCGAGCTGGACGGCGACGTAAACGGCCACAAGTTCAGCGTGTCC
GGCGAGGGCGAGGGCGATGCCACCTACGGCAAGCTGACCCTGAAGCTGATCTGCACCACCG
GCAAGCTGCCCGTGCCCTGGCCCACCCTCGTGACCACCCTGACCTGGGGCCTGCAGTGCTTC
GCCCGCTACCCCGACCACATGAAGCAGCACGACTTCTTCAAGTCCGCCATGCCCGAAGGCTA
CGTCCAGGAGCGCACCATCTTCTTCAAGGACGACGGCAACTACAAGACCCGCGCCGAGGTG
AAGTTCGAGGGCGACACCCTGGTGAACCGCATCGAGCTGAAGGGCATCGACTTCAAGGAGG
ACGGCAACATCCTGGGGCACAAGCTGGAGTACAACGCCATCAGCGACAACGTCTATATCAC
CGCCGACAAGCAGAAGAACGGCATCAAGGCCAACTTCAAGATCCGCCACAACATCGAGTGA
